# New Secondary Metabolites from the Marine-Derived Fungus *Talaromyces mangshanicus* BTBU20211089

**DOI:** 10.3390/md20020079

**Published:** 2022-01-18

**Authors:** Kai Zhang, Xinwan Zhang, Rui Lin, Haijin Yang, Fuhang Song, Xiuli Xu, Long Wang

**Affiliations:** 1 School of Light Industry, Beijing Technology and Business University, Beijing 100048, China; zhangkai2030302071@st.btbu.edu.cn; 2 School of Ocean Sciences, China University of Geosciences, Beijing 100083, China; zhangxinwan@cugb.edu.cn (X.Z.); linrui520@126.com (R.L.); yanghaijin52@163.com (H.Y.); 3 Key Laboratory of Mycology, Institute of Microbiology, Chinese Academy of Sciences, Beijing 100101, China

**Keywords:** marine-derived fungus, *Talaromyces mangshanicus*, antifungal, antibacterial

## Abstract

Seven new compounds, namely talaromanloid A (**1**), talaromydene (**2**), 10-hydroxy-8-demethyltalaromydine and 11-hydroxy-8-demethyltalaromydine (**3** and **4**), talaromylectone (**5**), and ditalaromylectones A and B (**6** and **7**), together with seven known compounds were identified from a marine-derived fungus, *Talaromyces mangshanicus* BTBU20211089, which was isolated from a sediment sample collected from the South China Sea. Their chemical structures were determined using spectroscopic data, including HRESIMS, 1D, and 2D NMR techniques. The absolute configurations of **1** and **2** were elucidated by comparing experimental and calculated ECD spectra. Compounds **1**, **2**, **6,** and **7** are new compounds possessing a novel carbon skeleton. Compound **6** is a dimeric molecule of **3** and **9**. Compound **7** shared a unique structure of the cyclized dimer of **3** and **4**. All the compounds were tested for their bioactivities against *Staphylococcus aureus*, *Escherichia coli*, and *Candida albicans*.

## 1. Introduction

Marine-derived fungi represent the most prolific source of new chemical entries with diverse bioactivities [1,2]. *Talaromyces* species had been included in the *Penicillium* subgenus *Biverticillium* and were classified as a valid genus by McNeill [3]. The *Talaromyces* fungus, widely distributed in marine and terrestrial environments, is an important natural resource producing enzymes and pigments of industrial importance, and sometimes can cause invasive mycosis. Different classes of secondary metabolites, such as polyene and isocoumarin [4], oxaones and oxaphenalenones [5], meroterpenoids [6], oxaphenalenone [7], diphenyl ether derivatives, and sesquiterpene-conjugated amino acids [8,9], have been characterized from marine-derived *Talaromyces* strains.

**Figure 1:**
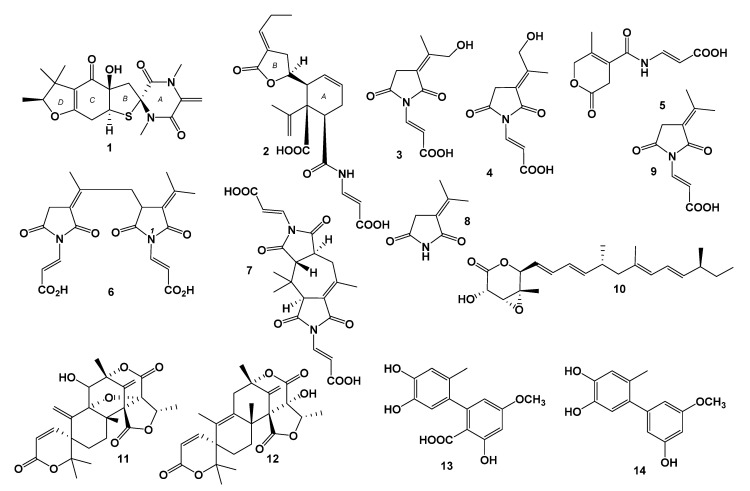
Chemical structures of **1**–**14**.

During our ongoing investigations into bioactive natural products from marine-derived microorganisms [10,11,12,13], a fungal strain of *Talaromyces mangshanicus* BTBU20211089, which was isolated from a sediment sample collected from the South China Sea, was found to be active against *Candida albicans.* Chemical investigation of this fungus cultured in rice solid media led to the isolation and identification of seven new compounds, namely, talaromanloid A (**1**), talaromydene (**2**), 10-hydroxy-8-demethyltalaromydine and 11-hydroxyd-8-emethyltalaromydine (**3** and **4**), talaromylectone (**5**), and ditalaromylectones A and B (**6** and **7**), together with seven known compounds. The known compounds were determined to be 3-(propan-2-ylidene)-pyrrolidine-2, 5-dione (**8**) [14], (*E*)-3-(2,5-dioxo-3-(propan-2-ylidene)pyrrolidin-1-yl)acrylic acid (**9**) [15], nafuredin (**10**) [16], dehydroaustinol (**11**) [17], austinolide (**12**) [18], altenusin (**13)**, and 5′-methoxy-6-methyl-biphenyl-3,4,3′-triol (**14**) [19] (Figure 1). Here, we report the isolation, structure elucidation, and bioactivities of these compounds.

## 2. Results

### 2.1. Structure Elucidation

**Figure 2:**
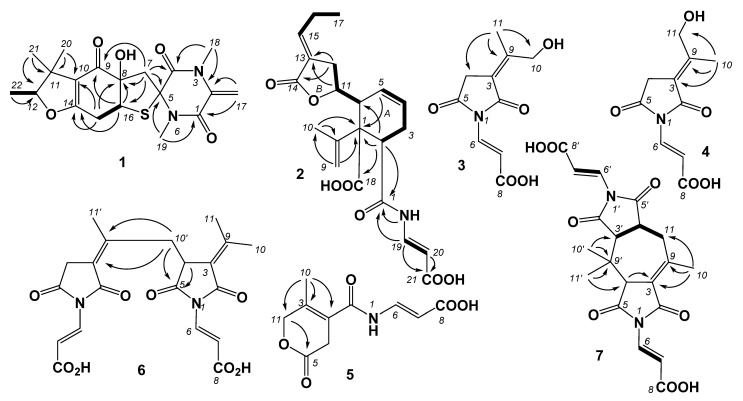
Key COSY (bold lines) and HMBC (arrows) correlations in **1**–**7**.

**Figure 3:**
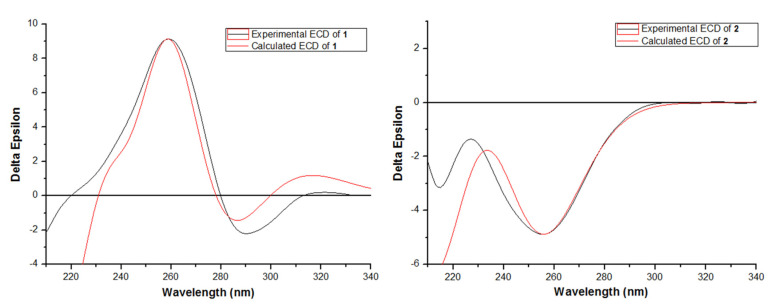
Calculated and experimental electronic circular dichroism (ECD) spectra of **1** and **2**.

Compound **1** was isolated as a light-yellow powder. The molecular formula of **1** was determined to be C_19_H_24_N_2_O_5_S based on the HRESIMS spectrum (*m/z* [M + H]^+^ 393.1481, calcd for C_19_H_25_N_2_O_5_S, 393.1479), accounting for nine degrees of unsaturation (Figure S1). The ^1^H NMR, ^13^C, and HSQC spectra of **1** (Figures S2–S4, Table 1) demonstrated signals for one terminal double bond (*δ*_H_ 5.57 (1H, H-17a) and 5.08 (1H, H-17b)/ *δ*_C_ 136.0 (C-3) and 102.8 (C-17)), two sp^3^ methylenes (*δ*_H_ 3.37 (1H, H-7a) and 2.86 (1H, H-7b)/*δ*_C_ 42.6 (C-7), 3.02 (1H, H-15a) and 2.47 (1H, H-15b)/*δ*_C_ 21.6 (C-15)), one sp^3^ oxygenated methine (*δ*_H_ 4.42 (1H, H-12)/*δ*_C_ 90.7 (C-12)), one sp^3^ methine (*δ*_H_ 4.18 (1H, H-16)/*δ*_C_ 50.9 (C-16)), four singlet methyl groups (*δ*_H_ 3.19 (3H, H-18)/*δ*_C_ 30.7 (C-18), *δ*_H_ 2.83 (3H, H-19)/*δ*_C_ 25.9 (C-19), *δ*_H_ 1.01 (3H, H-20)/*δ*_C_ 20.2 (C-20), *δ*_H_ 1.29 (3H, H-21)/*δ*_C_ 24.8 (C-21)), one doublet methyl (*δ*_H_ 1.28 (3H, H-22)/*δ*_C_ 13.9 (C-22)), one ketone carbonyl at *δ*_C_ 190.8 (C-9), two amide carbonyls at *δ*_C_ 158.0 (C-1) and 165.7 (C-3), one oxygenated sp^2^ quaternary carbon at *δ*_C_ 171.1 (C-14), two sp^2^ quaternary carbons at *δ*_C_ 136.0 (C-2) and 116.9 (C-10), and two sp^3^ quaternary carbons at *δ*_C_ 72.2 (C-5) and 81.6 (C-8). The downfield shift of oxygenated methine (C-12) in the ^13^C spectrum was consistent with that of phomalirazine [20]. Detailed analysis of the 2D NMR data (Figures S4–S6) confirmed the structure of **1**. The HMBC correlations (Figure 2) between H-17b and C-2, H_2_-17 and C-1, H_3_-18 and C-2 and C-4, and H_3_-19 and C-1 and C-5 revealed the *N*, *N*-dimethyldiketopiperazine moiety of ring A. The long-range HMBC correlations between H-15b and C-8, H_2_-15 and C-10 and C-14, and H-16 and C-8, C-9, and C-14 indicated the presence of the cyclopentenone moiety of ring C. The HMBC crossing peaks from H-7a to C-16, and H_2_-7 to C-4, C-5, C-8, and C-9 revealed that rings A and C were linked from C-5 to C-8 through C-7. The HMBC correlations between H_3_-20 and H_3_-21 and C-10, C-11, and C-12, and H_3_-22 and C-11 and C-12 indicated that the 1,1-dimethylpropyl was attached to C-10 by C-11. By analyzing the downfield chemical shifts of C-5, C-12, and C-14 and the molecular formula, C-5 and C-16 were linked by a sulfur atom to form the tetrahydrothiophene moiety of ring B, and C-12 and C-14 were linked by an oxygen atom to form the dihydrofuran moiety of ring D. Therefore, the planar structure of **1** was assigned. The absolute configurations of **1** were established as 5*S*, 8*R*, 12*S*, and 16*S* by comparing the experimental and calculated ECD spectra (Figure 3). Thus, the structure of **1** was determined and named talaromanloid A.

**Figure 4:**
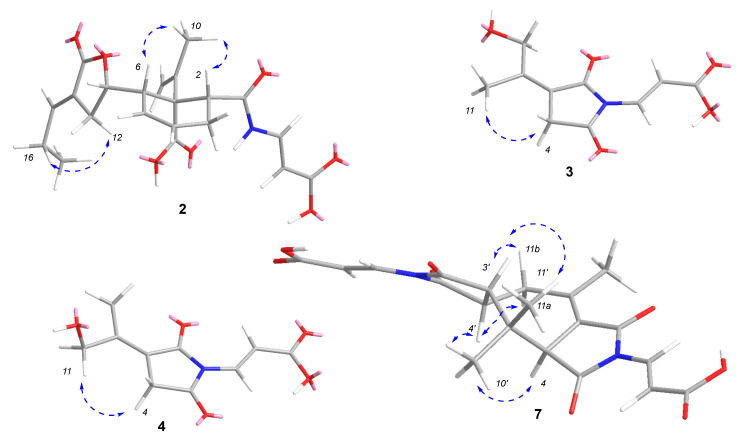
ROESY correlations in **2**–**4** and **7**.

Compound **2** was isolated as a light-yellow powder. The molecular formula of **2** was determined to be C_21_H_25_NO_7_ based on the HRESIMS spectrum (*m/z* [M + H]^+^ 404.1701, calcd for C_21_H_26_NO_7_, 404.1704), accounting for ten degrees of unsaturation (Figure S8). The ^1^H and ^13^C NMR spectra, along with HSQC data (Figures S9–S11, Table 1), revealed the presence of two methyls (*δ*_H_ 1.84/*δ*_C_ 19.6 (C-10); *δ*_H_ 0.98/*δ*_C_ 13.1 (C-17)), four methylenes (*δ*_H_ 2.34/*δ*_C_ 25.1 (C-3); *δ*_H_ 5.05, 4.68/*δ*_C_ 115.0 (C-9); *δ*_H_ 2.56/*δ*_C_ 27.7 (C-12); *δ*_H_ 2.13/*δ*_C_ 21.9 (C-16)), eight methines (*δ*_H_ 2.90/*δ*_C_ 44.7 (C-2); *δ*_H_ 6.05/*δ*_C_ 129.7 (C-4); *δ*_H_ 5.82/*δ*_C_ 123.1 (C-5); *δ*_H_ 3.20/*δ*_C_ 45.5 (C-6); *δ*_H_ 4.47/*δ*_C_ 80.3 (C-11); *δ*_H_ 6.77/*δ*_C_ 146.7 (C-15); *δ*_H_ 7.70/*δ*_C_ 137.9 (C-19); *δ*_H_ 5.49/*δ*_C_ 101.6 (C-20)), two sp^2^ quaternary carbons (*δ*_C_ 140.8 (C-8), 127.6 (C-13)), one two sp^3^ quaternary carbon (*δ*_C_ 57.9 (C-7)), and four carbonyls (*δ*_C_ 169.9 (C-1), 168.5 (C-14), *δ*_C_ 176.5 (C-18), *δ*_C_ 168.5 (C-21)). The ^1^H-^1^H COSY correlations (Figure S12**)** between H-2 and H_2_-3, H-4, H-5, H-6, H-11, and H_2_-12 revealed the connections of C-2, C-3, C-4, C-5, C-6, C-11, and C-12. The ^1^H-^1^H COSY crossing peaks from H-15 through H_2_-16 to H_3_-17 suggested a side chain of C-15/C-16/C-17. The ^1^H-^1^H COSY correlations between H-NH and H-19 and H-20, along with the coupling constants (*δ*_H_ 10.26, d (11.0), H-NH, 7.70 (dd, *J* = 14.0, 11.0 Hz, H-19), 5.49 (d, *J* = 14.0 Hz, H-20)) demonstrated the trans-double bond attached to the amino of N/C-19/C-20. In the HMBC spectrum (Figure S13), the correlations between H-2 and C-6 and C-7, and H-5 and C-7 suggested the presence of ring A. The HMBC correlations (Figure 2) between H_2_-9 and C-7 and C-10, and between H_3_-10 and C-7, C-8, and C-9 indicated the connection between C-8 and C-7. The carbonyls of C-1 and C-18 were confirmed by HMBC correlations between H-2 and C-1 and C-18, and between H-NH and C-1. The acrylic acid moiety attached to NH was characterized by HMBC correlations between H-19 and C-1 and between H-19 and H-20 and C-21. Ring B was revealed by the HMBC correlations between H-11 and C-13, and between H-12 and C-13 and C-14. The HMBC correlations between H_2_-16 and C-13 and C-15, together with the crossing peak from H-15 to C-14, indicated the connection between C-13 and C-15. The ROESY correlations (Figure 4 and Figure S14) between H_3_-10 (*δ*_H_ 1.84, s) and H-2 (*δ*_H_ 2.90, t, *J* = 8.5 Hz) and H-6 (*δ*_H_ 3.20, m) indicated that they were on the same side of ring A. The conformation of the double bond of C-13/C-15 was deduced by the ROESY correlations between H_2_-12 and H_2_-16 (Figure 2 and Figure S14). By comparing the experimental and calculated ECD spectra (Figure 3), the absolute configurations of **2** were established as 2*R*, 6*S*, 7*R*, 8S, and 11*S*. Thus, the structure of **2** was determined and named talaromydene.

Compound **3** was isolated as a colorless powder. The molecular formula of **3** was determined to be C_10_H_11_NO_5_ based on the HRESIMS spectrum (*m/z* [M + H]^+^ 226.0716, calcd for C_10_H_12_NO_5_, 226.0710), accounting for six degrees of unsaturation (Figure S15). The ^1^H and ^13^C NMR spectra, along with HSQC data (Figures S16–S18, Table 2), revealed the presence of one trans-double bond (*δ*_H_ 7.55 (d, *J* = 14.5 Hz)/*δ*_C_ 131.3 (C-6); 6.73 (d, *J* = 14.5 Hz)/*δ*_C_ 109.2 (C-7)), one sp^3^ methylene (*δ*_H_ 3.40/*δ*_C_ 33.6 (C-4)), one sp^3^ oxygenated methylene (*δ*_H_ 4.66/*δ*_C_ 60.0 (C-10)), one methyl group (*δ*_H_ 1.91/*δ*_C_ 18.1 (C-11)), two sp^2^ quaternary carbons (*δ*_C_ 117.8 (C-3); 156.2 (C-9)), and three carbonyls (*δ*_C_ 166.5 (C-2), 172.5 (C-5), 167.8 (C-8)). The NMR data were similar to those of (*E*)-3-(2,5-dioxo-3-(propan-2-ylidene) pyrrolidin-1-yl)acrylic acid [15], although one of the methyl groups was replaced by a hydroxymethyl in **3**. The hydroxyl of C-10 was confirmed by the HMBC correlations (Figure 2 and Figure S19) between H_3_-11 and C-3, C-9, and C-10, and ROESY correlation (Figure 4 and Figure S20) between H_3_-11 and H_2_-4. Thus, the structure of **3** was determined and named 10-hydroxy-8-demethyltalaromydine.

Compound **4** was isolated as a colorless powder. The molecular formula of **4** was determined to be C_10_H_11_NO_5_ based on the HRESIMS spectrum (*m/z* [M + H]^+^ 226.0716, calcd for C_10_H_12_NO_5_, 226.0710), accounting for six degrees of unsaturation (Figure S21). The ^1^H, ^13^C NMR, and HSQC data (Figures S22–S24, Table 2) shared high similarity with those of 3. The hydroxyl of C-11 was confirmed by the HMBC correlations (Figure 2 and Figure S25) between H_3_-10 and C-3, C-9, and C-11, and ROESY correlation (Figure 4 and Figure S26) between H_2_-11 and H_2_-4. Thus, the structure of **4** was determined and named 11-hydroxy-8-demethyltalaromydine.

Compound **5** was isolated as a colorless powder. The molecular formula of **5** was determined to be C_10_H_11_NO_5_ based on the HRESIMS spectrum (*m/z* [M + H]^+^ 226.0715, calcd for C_10_H_12_NO_5_, 226.0710), accounting for six degrees of unsaturation (Figure S27). The ^1^H, ^13^C, NMR, and HSQC data (Figures S28–S30, Table 2) shared high similarity with those of **3** and **4**. The signal at *δ*_H_ 10.59 (brs, H-NH) in the ^1^H spectrum and ^1^H-^1^H COSY correlation between H-NH and H-6 (*δ*_H_ 7.55 (br d, *J* = 8.5 Hz)) suggested that the pyrrolidine-2,5-dione in **5** was replaced by a ring-opening moiety. The HMBC correlations (Figure 2 and Figure S32) between H_2_-11 and C-5 (*δ*_C_ 168.6) revealed that C-5 and C-11 formed a lactone unit. Thus, the structure of **5** was determined and named talaromylectone.

Compound **6** was isolated as a light-yellow powder. The molecular formula of **6** was determined to be C_20_H_20_N_2_O_8_ based on the HRESIMS spectrum (*m/z* [M + H]^+^ 417.1287, calcd for C_20_H_21_N_2_O_8_, 417.1292), accounting for twelve degrees of unsaturation (Figure S33). The ^1^H and ^13^C NMR spectra, along with the HSQC data (Figures S34–S36, Table 3), revealed that **6** is a dimeric analog of **3** and (*E*)-3-(2,5-dioxo-3-(propan-2-ylidene) pyrrolidin-1-yl)acrylic acid (**9**). The proton NMR spectrum showed signals for three singlet methyl groups at *δ*_H_ 2.30 (H_3_-10), 2.03 (H_3_-11), and 1.95 (H_3_-11′), one singlet methylene at *δ*_H_ 3.46 (H_2_-4′), and one coupled methylene at *δ*_H_ 3.31 (dd, *J* = 13.0, 10.0 Hz, H-10′a) and 3.01 (dd, *J* = 13.0, 6.5 Hz, H-10′b) with one methine at *δ*_H_ 3.83 (dd, *J* = 10.0, 6.5 Hz, H-4), which revealed that one of the methyl groups in the monomer was replaced by methylene and attached to the methine of the other monomer. The linkage of C-4 and C-10′ was confirmed by the HMBC correlations (Figure 2 and Figure S37) between H_2_-10′ and C-3′ (*δ*_C_ 121.2) and C-9′ (*δ*_C_ 150.5), and between H_2_-10′ and H-4 and C-5 (***δ***_C_ 173.7). Therefore, the structure of **6** was determined and named ditalaromylectone A.

Compound **7** was isolated as a light-yellow powder. The molecular formula of **7** was determined to be C_20_H_20_N_2_O_8_ based on the HRESIMS spectrum (*m/z* [M + H]^+^ 417.1289, calcd for C_20_H_21_N_2_O_8_, 417.1292), accounting for twelve degrees of unsaturation (Figure S38). The ^1^H and ^13^C NMR spectra, along with the HSQC data (Figures S39–S41, Table 3), revealed that **7** is a dimeric analog of **4**, which possessed a different skeleton to that of **6**. The ^1^H-^1^H COSY correlations (Figure S42) between H_2_-11 and H-4′, and between H-3′ and H-4’, indicated the connection of C-11/C-4′/C-3′. The HMBC correlations (Figure 2 and Figure S43) between H-4 and C-3, H_3_-10 and C-3, C-9, and C-11, and H_3_-10′ and H_3_-11′ and C-4, C-3′, and C-9′ confirmed the presence of the cycloheptene moiety. In the ROESY spectra (Figure S44), the correlations between H-3′ (*δ*_H_ 2.90) and H-11b (*δ*_H_ 2.71)/H_3_-11′ (*δ*_H_ 1.06), between H-4′ (*δ*_H_ 3.62) and H-11a (*δ*_H_ 3.07)/H_3_-10’ (*δ*_H_ 1.31), and between H-4 (*δ*_H_ 3.70) and H_3_-10’ suggested the relative configurations of **7**. The optical rotation is near zero, so **7** was assigned as a racemic mixture and named ditalaromylectone B.

Seven known compounds, including 3-(propan-2-ylidene)-pyrrolidine-2, 5-dione (**8**), (E)-3-(2,5-dioxo-3-(propan-2-ylidene)pyrrolidin-1-yl)acrylic acid (**9**), nafuredin (**10**), dehydroaustinol (**11**), austinolide (**12**), altenusin (**13),** and 5′-methoxy-6-methyl-biphenyl-3,4,3′-triol (**14**), were identified by comparing the NMR data with the corresponding reported data.

**Figure 5:**
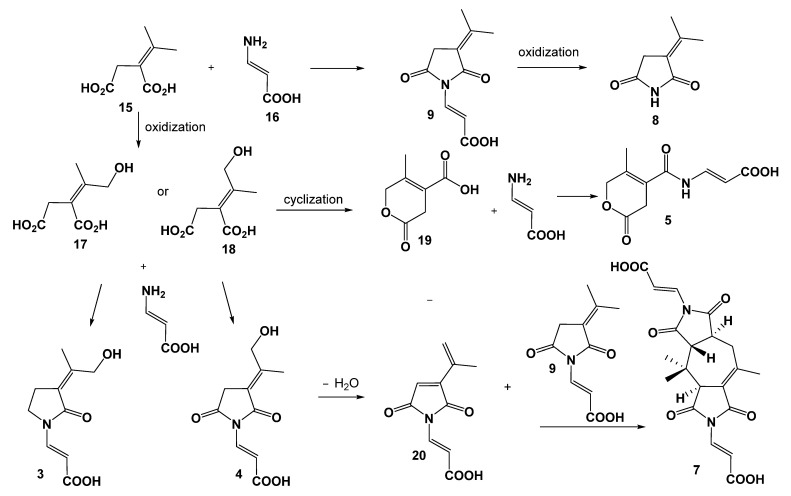
Plausible biosynthetic relationships of **3**–**5** and **7**–**9**.

The biosynthesis of **2**–**9** most likely proceeds via the same precursors, and plausible biosynthetic relationships of **3**–**5** and **7**–**9** are presented in Figure 5. Precursors **15** and **16** may be derived from the tricarboxylic acid cycle [21] and then form **9** by an amidation reaction**.** Compound **8** is produced by the oxidization of **9**. Compound **5** is proposed to be generated after the oxidization, cyclization, and amidation of **15** or **16**. Compound **7** is proposed to be derived from the cyclization of 9 and dehydration of **4**.

### 2.2. Biological Activity

These compounds were evaluated for their antibacterial activities against *Candida albicans* ATCC 10231, *Staphylococcus aureus* ATCC 25923, and *Escherichia coli* ATCC 25923. Compounds **6** and **13** showed an inhibitory effect against *C. albicans* with an MIC value of 200 μg/mL. Compounds **13** and **14** exhibited antibacterial activity against *S. aureus* with MIC values of 50 μg/mL.

## 3. Materials and Methods

### 3.1. General Experimental Procedures

Optical rotations ([α${]}_{D}^{25}$) were measured on an Anton Paar MCP 200 Modular Circular Polarimeter (Austria) in a 100 × 2 mm cell. CD spectra were recorded on an Applied Photophysics Chirascan spectropolarimeter (Surrey, UK). NMR spectra were obtained on a Bruker Avance 500 spectrometer with residual solvent peaks as references (DMSO-*d*_6_: *δ*_H_ 2.50, *δ*_C_ 39.52). High-resolution ESIMS measurements were obtained on an Accurate-Mass-Q-TOF LC/MS 6520 instrument (Santa Clara, CA, USA) in the positive ion mode. HPLC was performed using an Agilent 1200 Series separation module equipped with an Agilent 1200 Series diode array, Agilent 1260 Series fraction collector, and Agilent ZORBAX SB-C18 column (250 × 9.4 mm, 5 µm).

### 3.2. Microbial Material, Fermentation, Extraction and Purification

Strain *Talaromyces mangshanicus* BTBU20211089 was isolated from a mud sample collected from a sediment sample collected from the South China Sea and grown on a potato dextrose agar plate at 28 °C. The genomic DNA of BTBU20211089 was extracted using a Fungi Genomic DNA Extraction Kit (Solarbio Life Sciences, Beijing, China). The ITS region was amplified by using a conventional primer pair of ITS4 (5′ -TCCTCCGCTTATTGATATGC -3′) and ITS5 (5′-GGAAGTAAAAGTCGTAACAAGG -3′). PCR products were sent to Beijing Qingke Biotechnology Co., Ltd. (Beijing, China) for DNA sequencing and deposited in GenBank (accession number, OL905958). BTBU20211089 was identified as *Talaromyces mangshanicus* by comparing the internal transcribed spacer (ITS) region sequence with the GenBank database using the BLAST program. A neighbor-joining (NJ) tree (Figure S45) was constructed using the software package Mega version 5 [22]. The fungus was assigned the accession number BTBU20211089 in the culture collection at Beijing Technology and Business University, Beijing. The strain BTBU20211089 was inoculated on a potato dextrose agar plate and cultured for 7 days. Then, a slit of agar with the fungus was cut from the plate and inoculated in ten 1 L conical flasks, each containing a solid medium consisting of rice (200 g in 200 mL distilled water), and the flasks were incubated under static conditions at 28 °C for 30 days. The cultures were extracted three times with a mixture of EtOAc:MeOH (80:20), and the combined extracts were evaporated to dryness in vacuo. The residue was suspended in distilled water and partitioned with EtOAc. Then, the EtOAc layer was dried in vacuo to yield a dark residue (14.3 g). The EtOAc fraction was fractionated by vacuum liquid silica gel chromatography (80 × 80 mm column, Silica gel 60 H for thin-layer chromatography) using a stepwise gradient of hexane/CH_2_Cl_2_ (4:1, 7:3, 1:1, 1:4, 1:9, 3:97, and 0:100, *v/v*) and then a stepwise gradient of MeOH/CH_2_Cl_2_ (1:99, 2:98, 3:97, 5:95, 5:45, 1:4, and 100:0 *v/v*) to afford 14 fractions. Fraction G was purified by HPLC (Agilent ZORBAX SB-C18, 250 × 9.4 mm, 5 μm column, 3.0 mL/min, elution with 60% to 100% acetonitrile/H_2_O in 15 min) to yield **10** (5.8 mg). Fraction I was fractionated on a Sephadex LH-20 column using an isocratic elution of CH_2_Cl_2_:MeOH (2:1) to yield seven subfractions (I1–I7), and subfraction I2 was further purified by HPLC (Agilent ZORBAX SB-C18, 250 × 9.4 mm, 5 μm column, 3.0 mL/min, elution with 30% to 55% acetonitrile/H_2_O (0–20 min), and then to 80% acetonitrile/H_2_O (20–25 min)) to yield **1** (2.0 mg), **11** (10.4 mg), and **12** (14.3 mg). Subfraction I4 was further fractionated by HPLC (Agilent ZORBAX SB-C18, 250 × 9.4 mm, 5 μm column, 3.0 mL/min, elution with 30% to 100% acetonitrile/H_2_O) in 15 min to **8** (*R*_t_ 5.7 min, 3.6 mg) and **9** (20.5 mg). Fraction K was fractionated on a Sephadex LH-20 column using an isocratic elution of CH_2_Cl_2_:MeOH (2:1) to yield eight subfractions (K1–K8). Subfraction K3 was further fractionated by HPLC (Agilent ZORBAX SB-C18, 250 × 9.4 mm, 5 μm column, 3.0 mL/min, with 30% to 55% acetonitrile/H_2_O) in 15 min to yield **2** (*R*_t_ 13.4 min, 3.5 mg). Subfraction K4 was further fractionated by HPLC (Agilent ZORBAX SB-C18, 250 × 9.4 mm, 5 μm column, 3.0 mL/min, elution with 30% to 100% acetonitrile/H_2_O) in 15 min to yield **6** (1.7 mg). Subfraction K5 was further fractionated by HPLC (Agilent ZORBAX SB-C18, 250 × 9.4 mm, 5 μm column, 3.0 mL/min, elution with 30% acetonitrile/H_2_O) in 15 min to yield **7** (1.8 mg). Subfraction K6 was further fractionated by HPLC (Agilent ZORBAX SB-C18, 250 × 9.4 mm, 5 μm column, 3.0 mL/min, elution with 10% to 27% acetonitrile/H_2_O) in 15 min to yield **3** (1.8 mg), **4** (1.2 mg), and **5** (3.3 mg). Subfraction K8 was further fractionated by HPLC (Agilent ZORBAX SB-C18, 250 × 9.4 mm, 5 μm column, 3.0 mL/min, elution with 40% to 50% acetonitrile/H_2_O) in 15 min to yield **13** (6.8 mg) and **14** (5.6 mg).

Talaromanloid A (**1**): Light-yellow powder; [α${]}_{D}^{25}$ + 43.0 (*c* 0.1, MeOH); CD (*c* 5.0 × 10^−5^, CH_3_OH), λ_max_(∆ε) 259 nm (+9.14) and 290 nm (−2.20); ^1^H and ^13^C NMR data, Table 1; HRESIMS *m/z* 393.1481 [M + H]^+^ (calcd for C_19_H_25_N_2_O_5_S, 393.1479).

Talaromydene (**2**): Light-yellow powder; [α${]}_{D}^{25}$ −67.0 (*c* 0.1, MeOH); CD (*c* 5.0 × 10^−5^, CH_3_OH), λ_max_(∆ε) 256 nm (−4.88); ^1^H and ^13^C NMR data, Table 1; HRESIMS *m/z* 404.1701 [M + H]^+^ (calcd for C_21_H_26_NO_7_, 404.1704).

10-Hydroxydemethyltalaromydine (**3**): Colorless powder; ^1^H and ^13^C NMR data, Table 2; HRESIMS *m/z* 226.0716 [M + H]^+^ (calcd for C_10_H_12_NO_5_, 226.0710).

11. -hydroxydemethyltalaromydine (**4**): Colorless powder; ^1^H and ^13^C NMR data, Table 2; HRESIMS *m/z* 226.0716 [M + H]^+^ (calcd for C_10_H_12_NO_5_, 226.0710).

Talaromylectone (**5**): Colorless powder; ^1^H and ^13^C NMR data, Table 2; HRESIMS *m/z* 226.0715 [M + H]^+^ (calcd for C_10_H_12_NO_5_, 226.0710).

Ditalaromylectone A (**6**): Light-yellow powder; [α${]}_{D}^{25}$ +6.0 (*c* 0.1, MeOH); ^1^H and ^13^C NMR data, Table 3; HRESIMS *m/z* 417.1287 [M + H]^+^ (calcd for C_20_H_21_N_2_O_8_, 417.1292).

Ditalaromylectone B (**7**): Light-yellow powder; [α${]}_{D}^{25}$ 0.0 (*c* 0.1, MeOH); ^1^H and ^13^C NMR data, Table 3; HRESIMS *m/z* 417.1289 [M + H]^+^ (calcd for C_20_H_21_N_2_O_8_, 417.1292).

### 3.3. ECD Computation Method

Conformation searching was performed using OpenBabel by a genetic algorithm (GA) with the default settings [23]. The conformers were subsequently optimized using the DFT method at the B3LYP/TZVP level with GAUSSIAN 09 [24]. The TDDFT calculations of their low-energy conformations within 0–2.5 kcal/mol were performed at the same level with 40 single excited states. The solvent effect was taken into account by using the polarizable continuum model (PCM).

### 3.4. Biological Activity

Compounds **1**–**14** were evaluated for their antimicrobial activities in 96 well plates according to the Antimicrobial Susceptibility Testing Standards outlined by the Clinical and Laboratory Standards Institute document M07-A7 (CLSI) and our previous report [13]. The MIC was defined as the minimum concentration of the compound that prevented visible growth of the microbes.

## 4. Conclusions

Seven new compounds, talaromanloid A (**1**), talaromydene (**2**), 10-hydroxy-8-demethyltalaromydine and 11-hydroxy-8-demethyltalaromydine (**3** and **4**), talaromylectone (**5**), and ditalaromylectones A and B (**6** and **7**), and seven known compounds (**8**–**14**) were isolated from the marine-derived fungus *Talaromyces mangshanicus* BTBU20211089. The structures of the new compounds were elucidated by detailed spectroscopic analysis. The absolute configurations of **1** and **2** were elucidated by comparing experimental and calculated ECD spectra. Compound **6** was a dimeric molecule of **3** and **9** possessing a novel carbon skeleton. Compound **7** possessed a unique novel carbon skeleton structure of a cyclized dimer of **3** and **4**. Compounds **6** and **13** showed an inhibitory effect against *C. albicans* with an MIC value of 200 μg/mL. Compounds **13** and **14** exhibited antibacterial activity against *S. aureus* with MIC values of 50 μg/mL.

## Figures and Tables

**Table 1:** ^1^H (500 MHz) and ^13^C NMR (125 MHz) data of **1** and **2** (DMSO-*d*_6_).

Position	1		2	
	*δ*_C_, Type	*δ*_H_ (*J* in Hz)	*δ*_C_, Type	*δ*_H_ (*J* in Hz)
1	158.0, C		169.9, C	
2	136.0, C		44.7, CH	2.90, t (8.5)
3			25.1, CH_2_	2.34, m
4	165.7, C		129.7, CH	6.05, m
5	72.2, C		123.1, CH	5.82, dd (10.0, 1.5)
6			45.5, CH	3.20, m
7	42.6, CH_2_	3.37, d (14.5) 2.86, d (14.5)	57.9, C	5.05, s
8	81.6, C		140.8, C	
9	190.8, C		115.0, CH_2_	5.05, s 4.68, s
10	116.9, C		19.6, CH_3_	1.84, s
11	42.7, C		80.3, CH	4.47, ddd (10.0, 4.0, 4.0)
12	90.7, CH	4.42, q (7.0)	27.7, CH_2_	2.56, m
13			127.6, C	
14	171.1, C		168.5, C	
15	21.6, CH_2_	3.02, dd (19.5, 6.0) 2.47, overlap	146.7, CH	6.77, t (7.5)
16	50.9	4.18, dd (6.0, 1.0)	21.9, CH_2_	2.13, dq (7.5, 7.5)
17	102.8, CH_2_	5.57, d (1.0) 5.08, d (1.0)	13.1, CH_3_	0.98, t (7.5)
18	30.7, CH_3_	3.19, s	176.5, C	
19	29.5, CH_3_	2.83, s	137.9, CH	7.70, dd (14.0, 11.0)
20	20.2, CH_3_	1.01, s	101.6, CH	5.49, d (14.0)
21	24.8, CH_3_	1.29, s	168.5, C	
22	13.9, CH_3_	1.28, s (5.5)		
NH				10.26, d (11.0)

**Table 2:** ^1^H (500 MHz) and ^13^C NMR (125 MHz) data of **3**–**5** (DMSO-*d*_6_).

Position	3		4		5	
	*δ*_C_, Type	*δ*_H_, (*J* in Hz)	*δ*_C_, Type	*δ*_H_, (*J* in Hz)	*δ*_C_, Type	*δ*_H_, (*J* in Hz)
2	166.5, C		167.4, C		164.7, C	
3	117.8, C		117.2, C		121.9, C	
4	33.6, CH_2_	3.40, br s	32.9, CH_2_	3.41, br d, 1.0	30.7, CH_2_	3.29, br q (2.0)
5	172.5, C		172.5, C		168.6, C	
6	131.3, CH	7.55, d (14.5)	131.3, CH	7.57, d (15.0)	137.8, CH	7.81, br d (8.5)
7	109.2, CH	6.73, d (14.5)	109.0, CH	6.74, d (15.0)	102.3, CH	5.56, d (14.0)
8	167.8, C		167.8, C		168.2, C	
9	156.2, C		154.3, C		136.6, C	
10	60.0, CH_2_	4.66, s	15.5, CH_3_	2.27, s	16.0, CH_3_	1.88, s
11	18.1, CH_3_	1.91, s	63.4, CH_2_	4.05, s	71.2, CH_2_	4.83, br s
NH						10.59, br s

**Table 3:** ^1^H (500 MHz) and ^13^C NMR (125 MHz) data of **6** and **7** (DMSO-*d*_6_).

Position	6		7	
	*δ*_C_, Type	*δ*_H_, mult (*J* in Hz)	*δ*_C_, Type	*δ*_H_, mult (*J* in Hz)
2	166.8, C		166.5, C	
3	122.1, C		119.0, C	
4	42.1, CH	3.83, dd (10.0, 6.5)	49.9, CH	3.70, s
5	173.7, C		173.3, C	
6	131.0, CH	7.53, d (15.0)	130.7, CH	7.57, d (15.0)
7	109.6, CH	6.70, d (15.0)	110.0, CH	6.77, d (15.0)
8	167.7, C		167.5, C	
9	153.7, C		155.6, C	
10	21.1, CH_3_	2.30, s	21.2, CH_3_	2.31, s
11	23.8, CH_3_	2.03, s	37.8, CH_2_	3.07, dd (19.5, 7.5) 2.71, dd (19.5, 11.0)
2′	166.3, C		174.5, C	
3′	121.2, C		52.0, CH	2.90, d (8.5)
4′	33.8, CH_2_	3.46, s	36.7, CH	3.62, m
5′	172.0, C		175.9, C	
6′	131.0, CH	7.51, d (15.0)	130.7, CH	7.46, d (15.0)
7′	109.6, CH	6.61, d (15.0)	109.8, CH	6.70, d (15.0)
8′	166.7, C		167.5, C	
9′	150.5, C		39.1, C	
10′	35.4, CH_2_	3.31, dd (13.0, 10.0) 3.01, dd (13.0, 6.5)	20.4, CH_3_	1.31, s
11′	22.5, CH_3_	1.95, s	22.1, CH_3_	1.06, s

## Data Availability

Not applicable.

## Supplementary Materials

The following supporting information can be downloaded at https://www.mdpi.com/article/10.3390/md20020079/s1: Figures S1–S44: HRESIMS, 1D and 2D NMR for compounds 1–7; Figure S45: Neighbor-joining phylogenetic tree of strain BTBU20211089.
