# Supplementary material for: New Secondary Metabolites from the Marine-Derived Fungus Talaromyces mangshanicus BTBU20211089
Source: Mar Drugs. 2022 Jan 18;20(2):79. doi: 10.3390/md20020079 (PMC8879399; doi:10.3390/md20020079)
Supplement: Supplementary file 1 [file marinedrugs-20-00079-s001.zip › marinedrugs-1547805-supplementary.pdf]

SUPPLEMENTARY MATERIAL

New Secondary Metabolites the Marine-derived Fungus *Talaromyces mangshanicus* BTBU20211089

Kai Zhang [1,†], Xinwan Zhang [2,†], Rui Lin [2], Haijin Yang [2], Fuhang Song [1,*], Xiuli Xu [2,*] and Long Wang [3,*]

1 School of Light Industry, Beijing Technology and Business University, Beijing 100048, China; zhangkai2030302071@st.btbu.edu.cn
2 School of Ocean Sciences, China University of Geosciences, Beijing 100083, China; zhangxinwan@cugb.edu.cn (X.Z.); linrui520@126.com (R.L.); yanghaijin52@163.com (H.Y.)
3 Key Laboratory of Mycology, Institute of Microbiology, Chinese Academy of Sciences, Beijing 100101, China
* Correspondence: songfuhang@btbu.edu.cn (F.S.); xuxl@cugb.edu.cn (X.X.); wl_dgk@im.ac.cn (L.W.)
† These authors contributed equally to this work.

Table of Contents

Figure S1. HRESIMS spectrum for **1** 4

Figure S2. ^{1}H NMR spectrum (500 MHz, DMSO-d_6) of **1** 4

Figure S3. ^{13}C NMR spectrum (125 MHz, DMSO-d_6) of **1** 5

Figure S4. HSQC spectrum (500 MHz, DMSO-d_6) of **1** 5

Figure S5. ^{1}H -^{1}H COSY spectrum (500 MHz, DMSO-d_6) of **1** 6

Figure S6. HMBC spectrum (500 MHz, DMSO-d_6) of **1** 6

Figure S7. ROESY spectrum (500 MHz, DMSO-d_6) of **1** 7

Figure S8. HRESIMS spectrum for **2** 7

Figure S9. ^{1}H NMR spectrum (500 MHz, DMSO-d_6) of **2** 8

Figure S10. ^{13}C NMR spectrum (125 MHz, DMSO-d_6) of **2** 8

Figure S11. HSQC spectrum (500 MHz, DMSO-d_6) of **2** 9

Figure S12. ^{1}H -^{1}H COSY spectrum (500MHz, DMSO-d_6) of **2** 9

Figure S13. HMBC spectrum (500MHz, DMSO) of **2** 10

Figure S14. ROESY spectrum (500MHz, DMSO) of **2** 10

Figure S15. HRESIMS spectrum for **3** 11

Figure S16. ^{1}H NMR spectrum (500 MHz, DMSO-d_6) of **3** 11

Figure S17. ^{13}C NMR spectrum (125 MHz, DMSO-d_6) of **3** 12

Figure S18. HSQC spectrum (500 MHz, DMSO-d_6) of **3** 12

Figure S19. HMBC spectrum (500 MHz, DMSO-d_6) of **3** 13

Figure S20. ROESY spectrum (500 MHz, DMSO-d_6) of **3** 13

Figure S21. HRESIMS spectrum for **4** 14

Figure S22. ^{1}H NMR spectrum (500 MHz, DMSO-d_6) of **4** 14

Figure S23. ^{13}C NMR spectrum (125 MHz, DMSO-d_6) of **4** 15

Figure S24. HSQC spectrum (500 MHz, DMSO-d_6) of **4** 15

Figure S25. HMBC spectrum (500 MHz, DMSO-d_6) of **4** 16

Figure S26. ROESY spectrum (500 MHz, DMSO-d_6) of **4** 16

Figure S27. HRESIMS spectrum for **5** 17

Figure S28. ^{1}H NMR spectrum (500 MHz, DMSO-d_6) of **5** 17

Figure S29. ^{13}C NMR spectrum (125 MHz, DMSO-d_6) of **5** 18

Figure S30. HSQC spectrum (500 MHz, DMSO-d_6) of **5** 18

Figure S31. ^1H -^1H COSY spectrum (500 MHz, DMSO-d_6) of **5** 19
Figure S32. HMBC spectrum (500 MHz, DMSO-d_6) of **5** 19
Figure S33. HRESIMS spectrum for **6** 20
Figure S34. ^1H NMR spectrum (500 MHz, DMSO-d_6) of **6** 20
Figure S35. ^{13}C NMR spectrum (125 MHz, DMSO-d_6) of **6** 21
Figure S36. HSQC spectrum (500 MHz, DMSO-d_6) of **6** 21
Figure S37. HMBC spectrum (500 MHz, DMSO-d_6) of **6** 22
Figure S38. HRESIMS spectrum for **7** 22
Figure S39. ^1H NMR spectrum (500 MHz, DMSO-d_6) of **7** 23
Figure S40. ^{13}C NMR spectrum (125 MHz, DMSO-d_6) of **7** 23
Figure S41. HSQC spectrum (500 MHz, DMSO-d_6) of **7** 24
Figure S42. ^1H -^1H COSY spectrum (500 MHz, DMSO-d_6) of **7** 24
Figure S43. HMBC spectrum (500 MHz, DMSO-d_6) of **7** 25
Figure S44. ROESY spectrum (500 MHz, DMSO-d_6) of **7** 25
Figure S45. Neighbor-joining phylogenetic tree of strain BTBU20211089 26

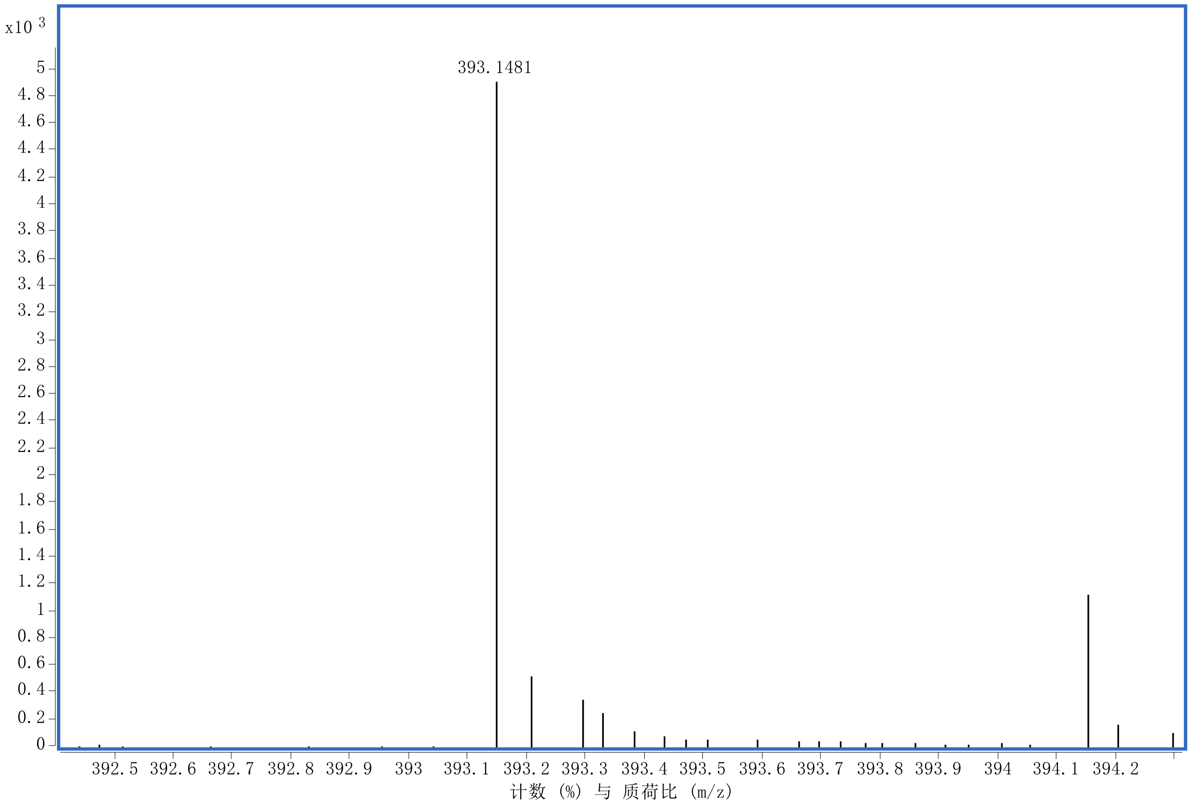

Figure S1. HRESIMS spectrum for compound **1**

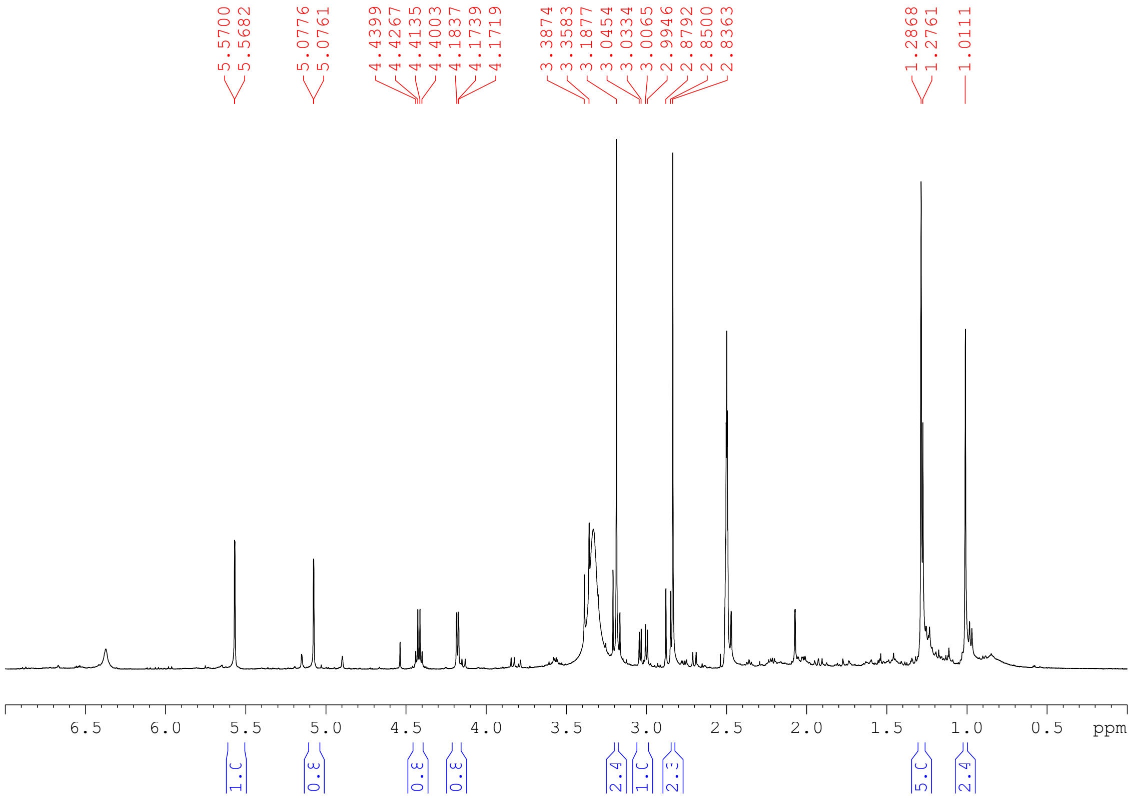

Figure S2. ^{1}H NMR spectrum (500 MHz, DMSO-d_6) of **1**

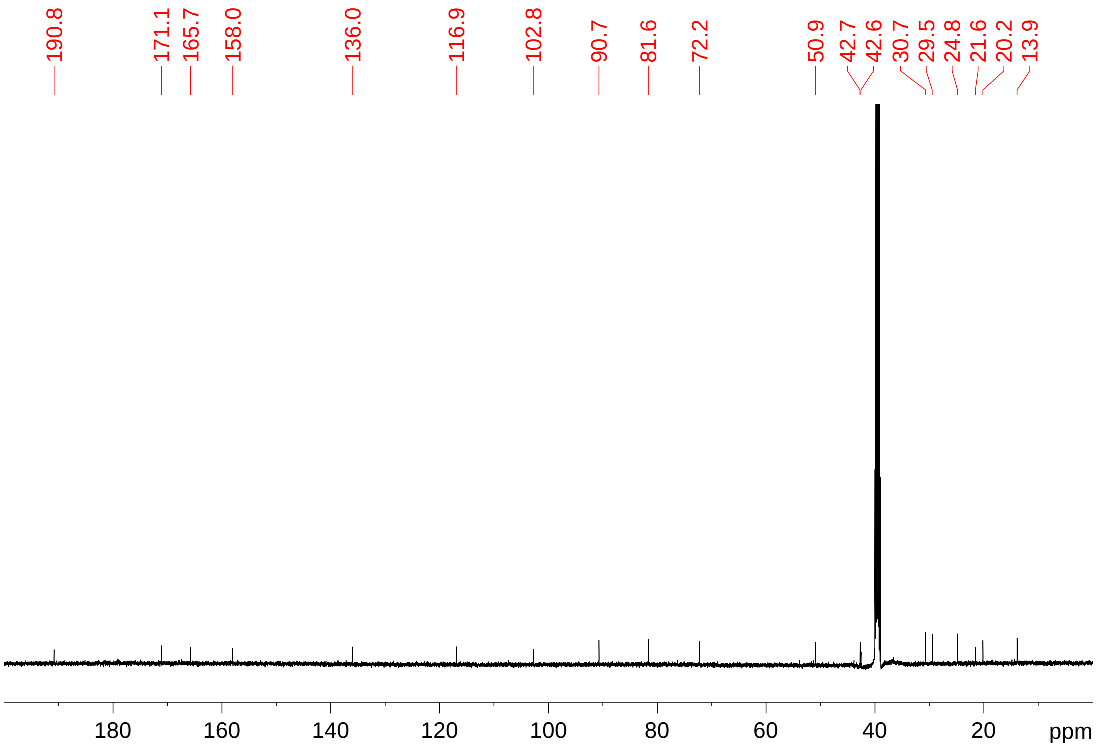

Figure S3. ^{13}C NMR spectrum (125 MHz, DMSO-d_6) of **1**

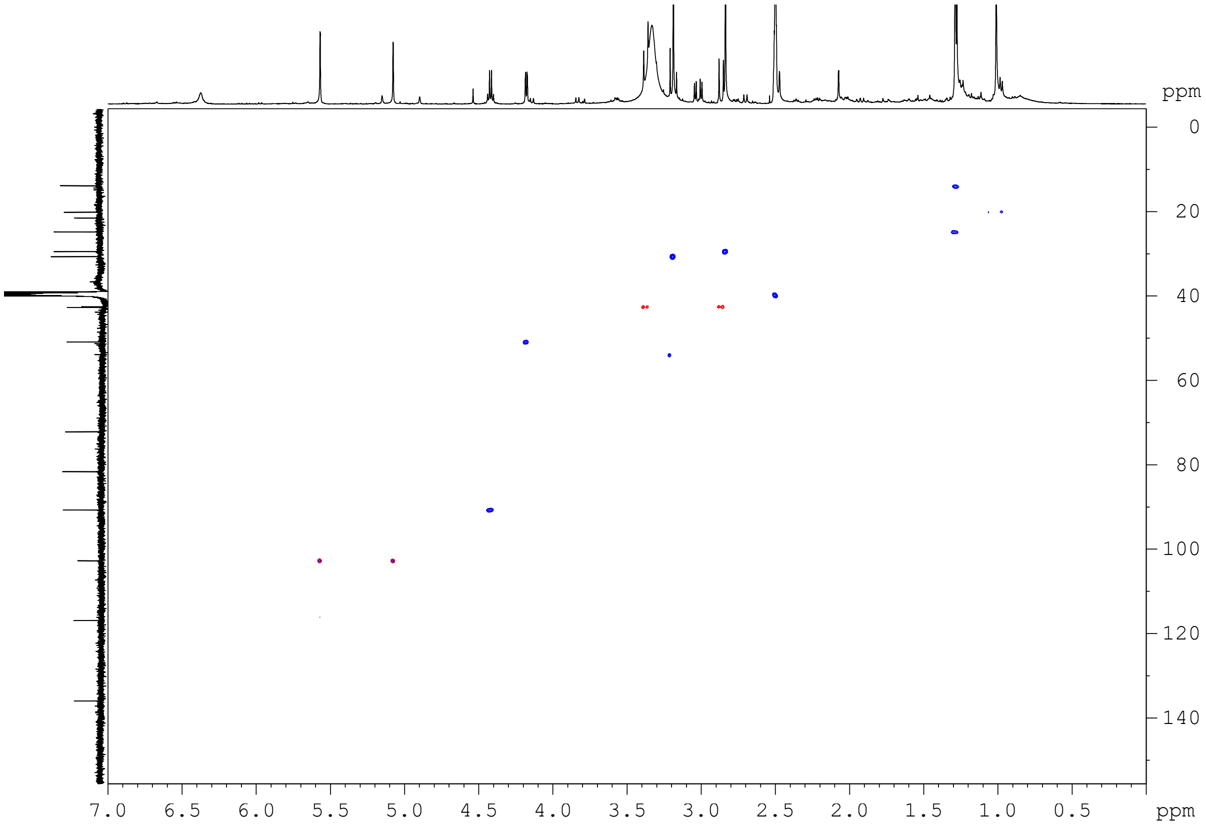

Figure S4. HSQC spectrum (500 MHz, DMSO-d_6) of **1**

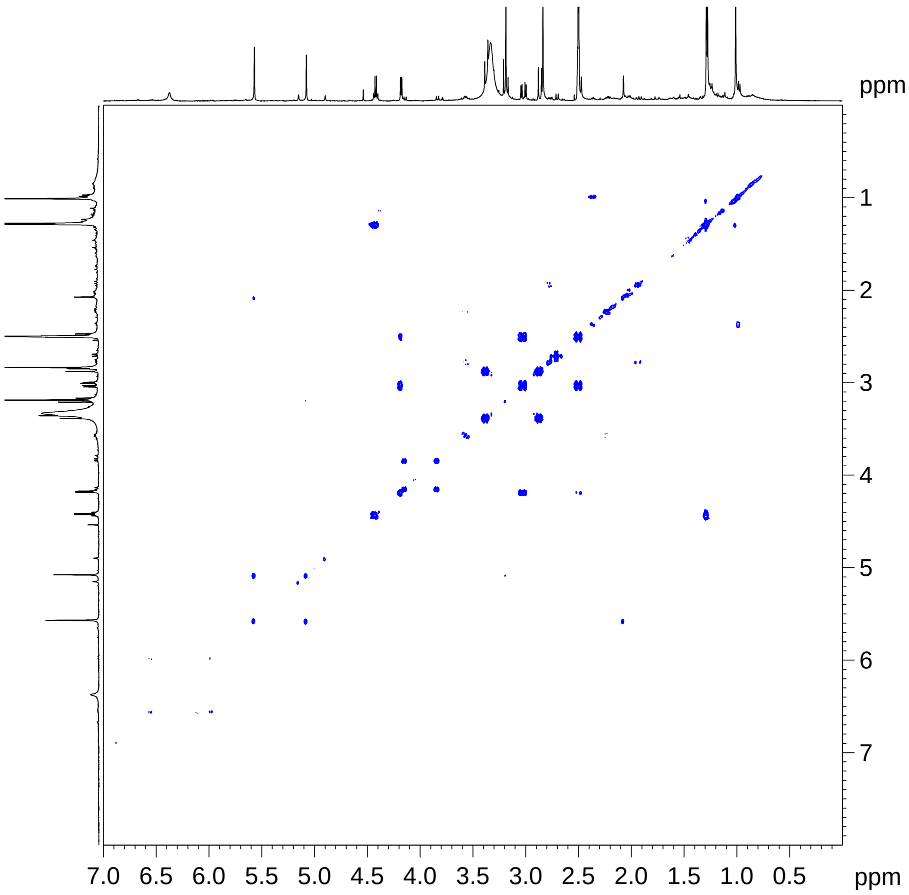

Figure S5. ^{1}H -^{1}H COSY spectrum (500 MHz, DMSO-d_6) of **1**

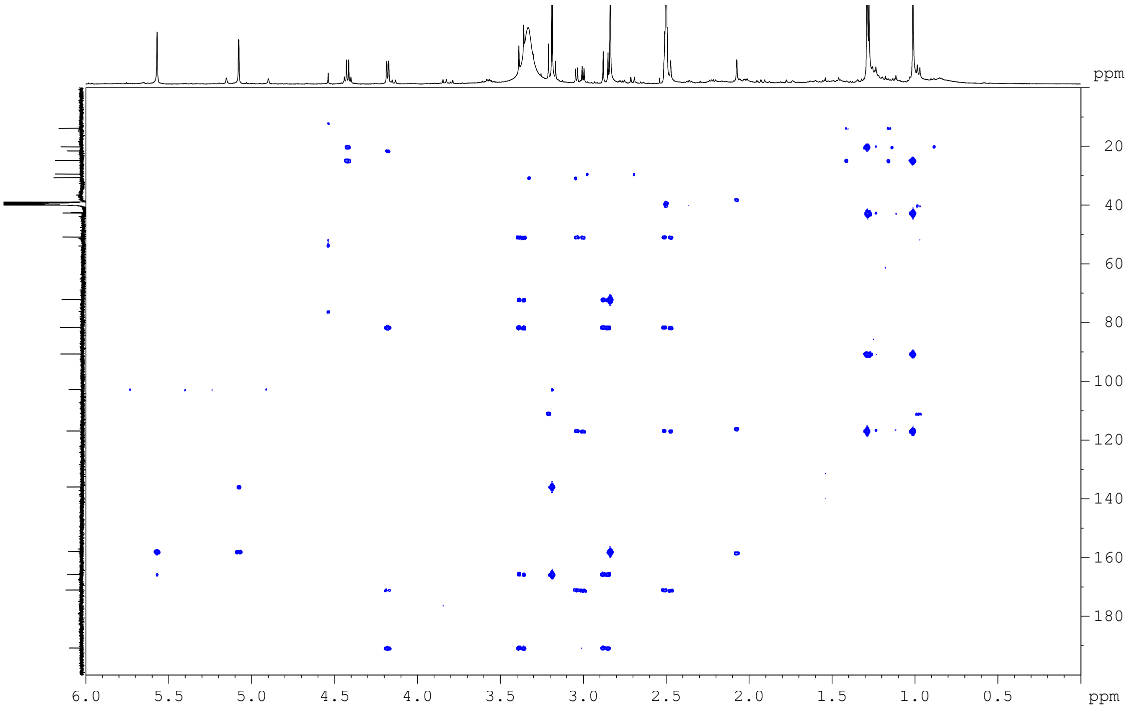

Figure S6. HMBC spectrum (500 MHz, DMSO-d_6) of **1**

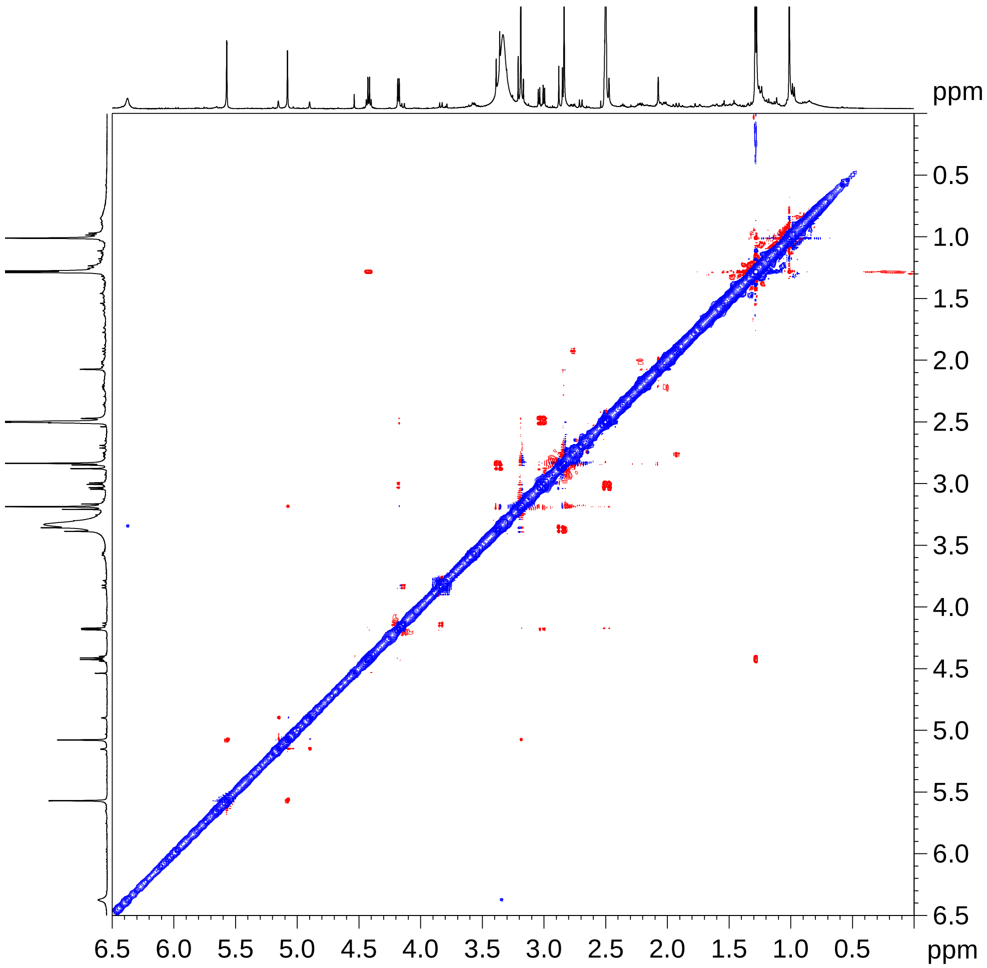

Figure S7. ROESY spectrum (500 MHz, DMSO-d_6) of **1**

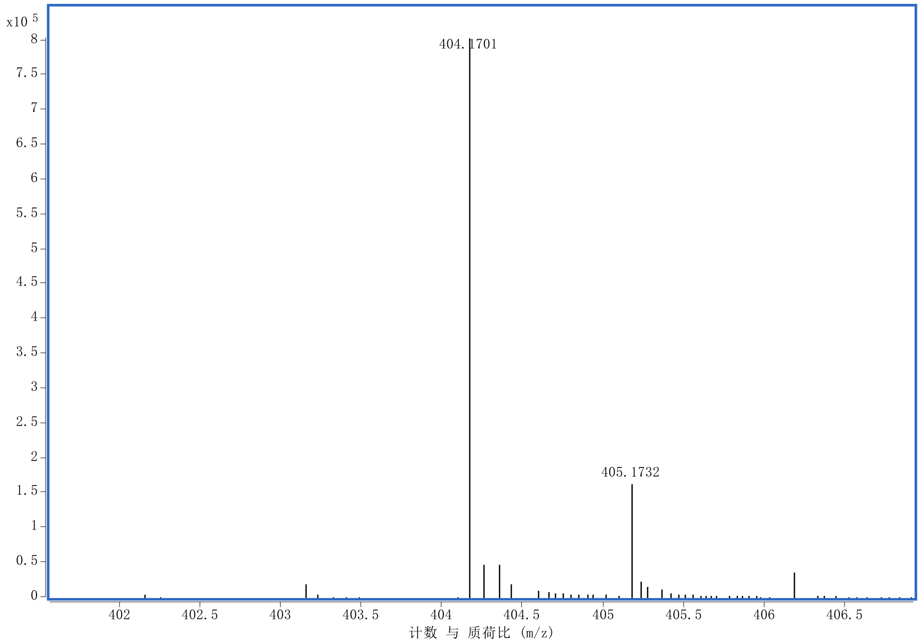

Figure S8. HRESIMS spectrum for **2**

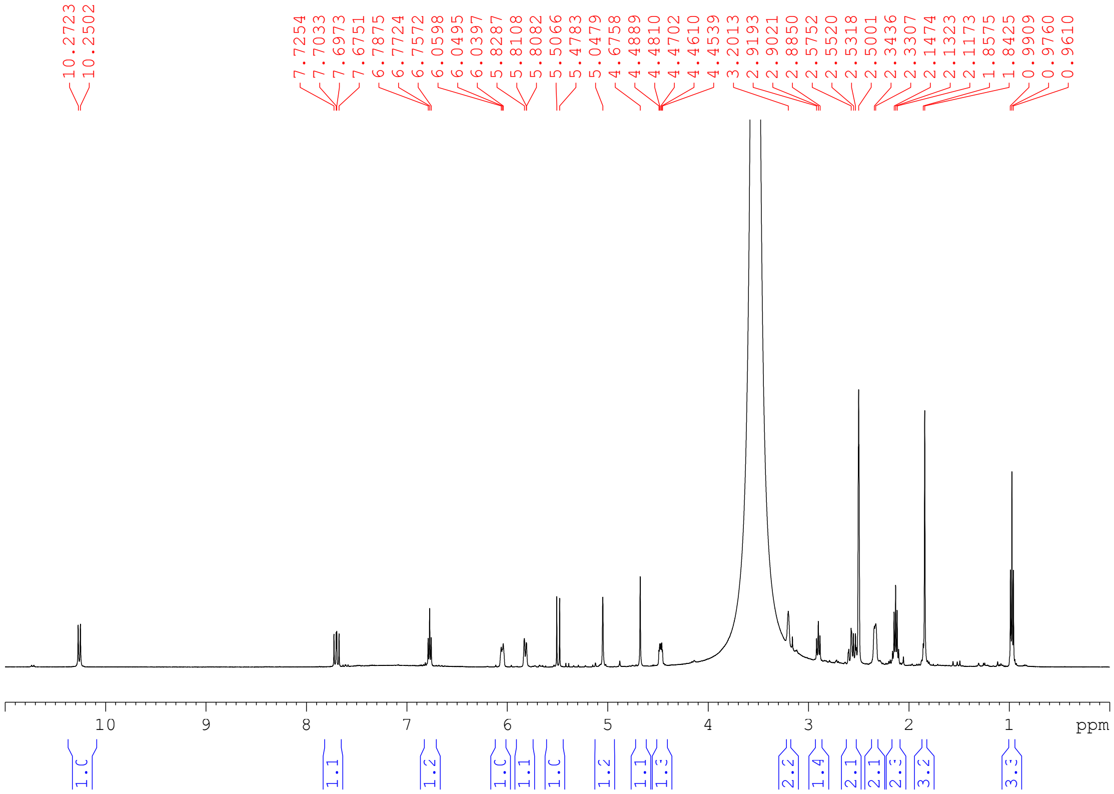

Figure S9. ^{1}H NMR spectrum (500 MHz, DMSO-d_6) of **2**

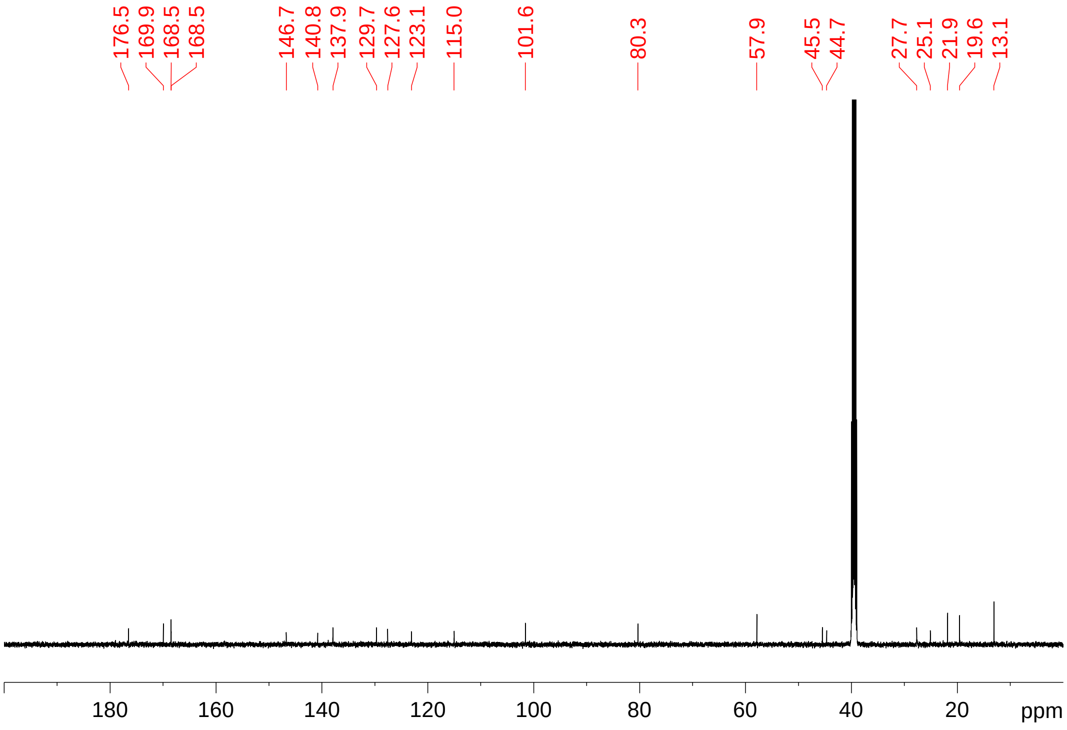

Figure S10. ^{13}C NMR spectrum (125 MHz, DMSO-d_6) of **2**

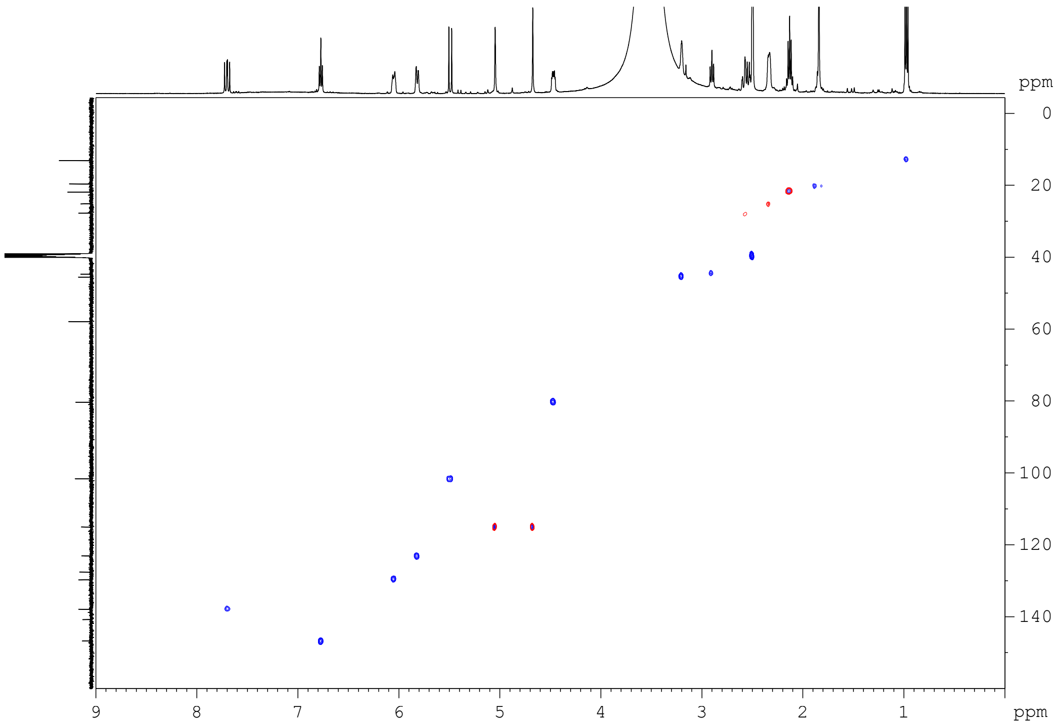

Figure S11. HSQC spectrum (500 MHz, DMSO-d_6) of **2**

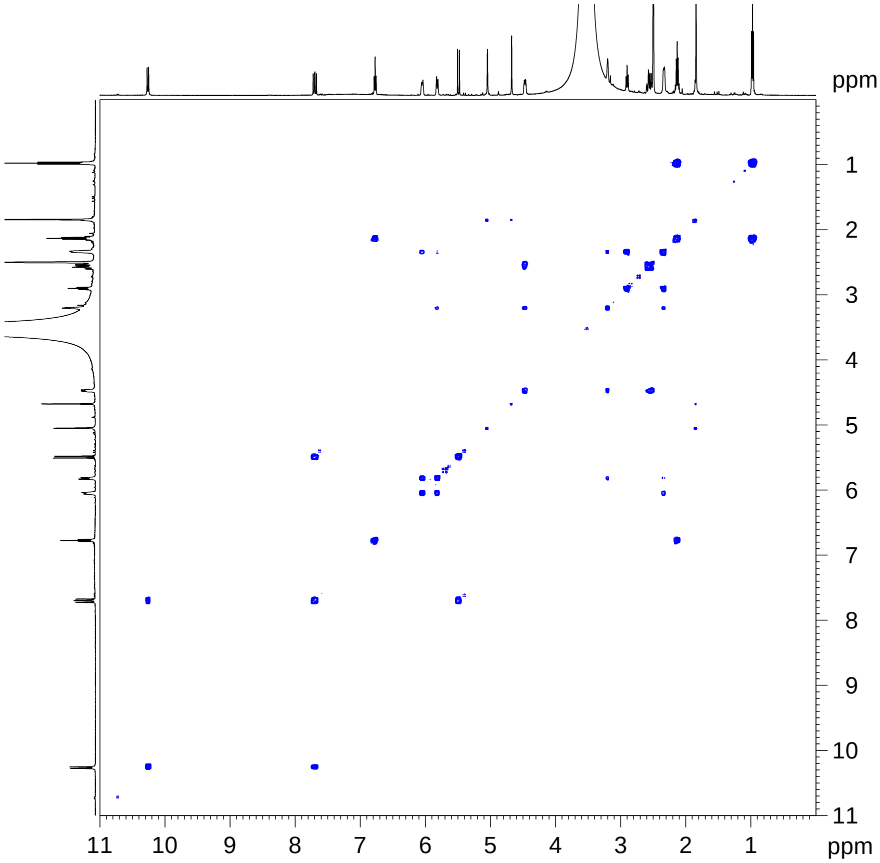

Figure S12. ^{1}H -^{1}H COSY spectrum (500MHz, DMSO-d_6) of **2**

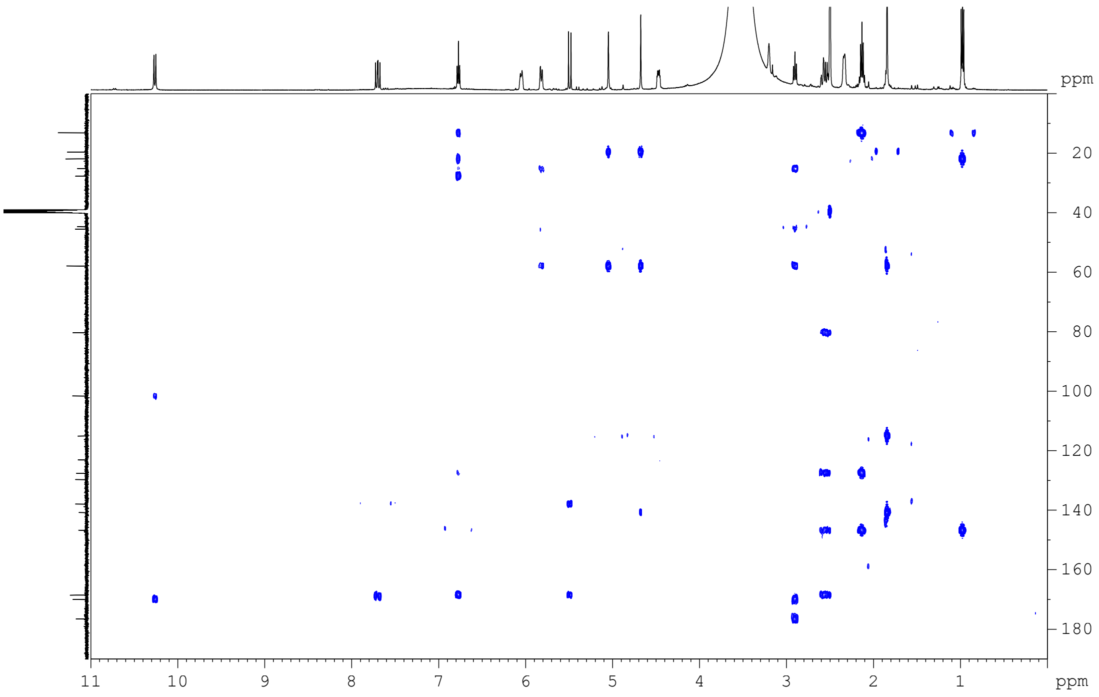

Figure S13. HMBC spectrum (500 MHz, DMSO-d_6) of **2**

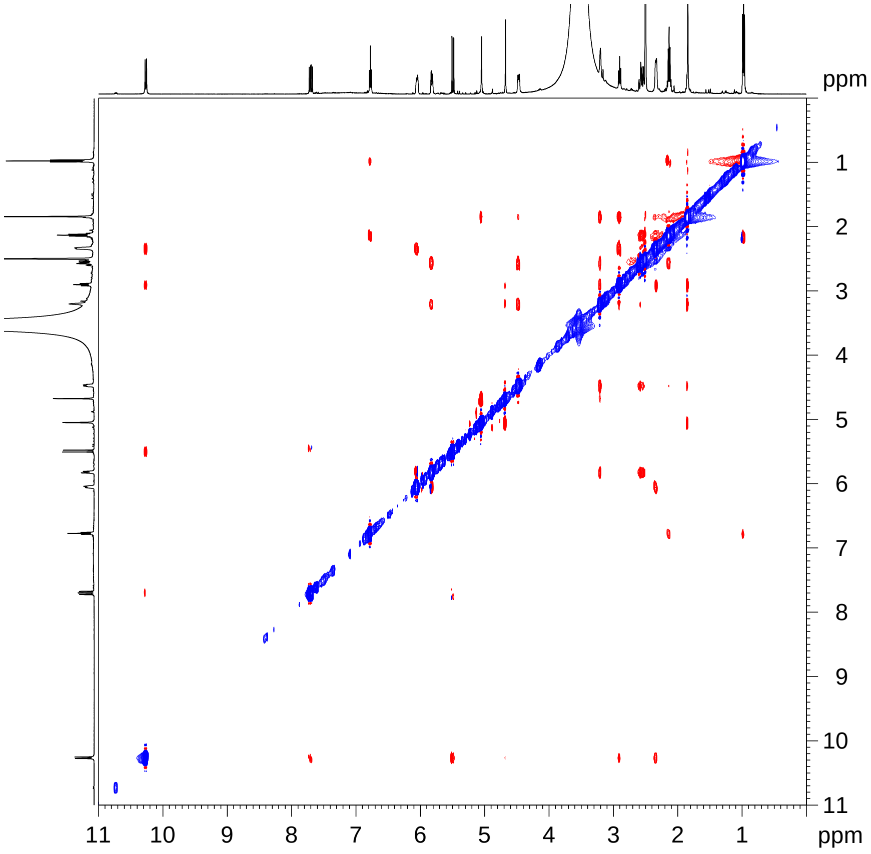

Figure S14. ROESY spectrum (500 MHz, DMSO-d_6) of **2**

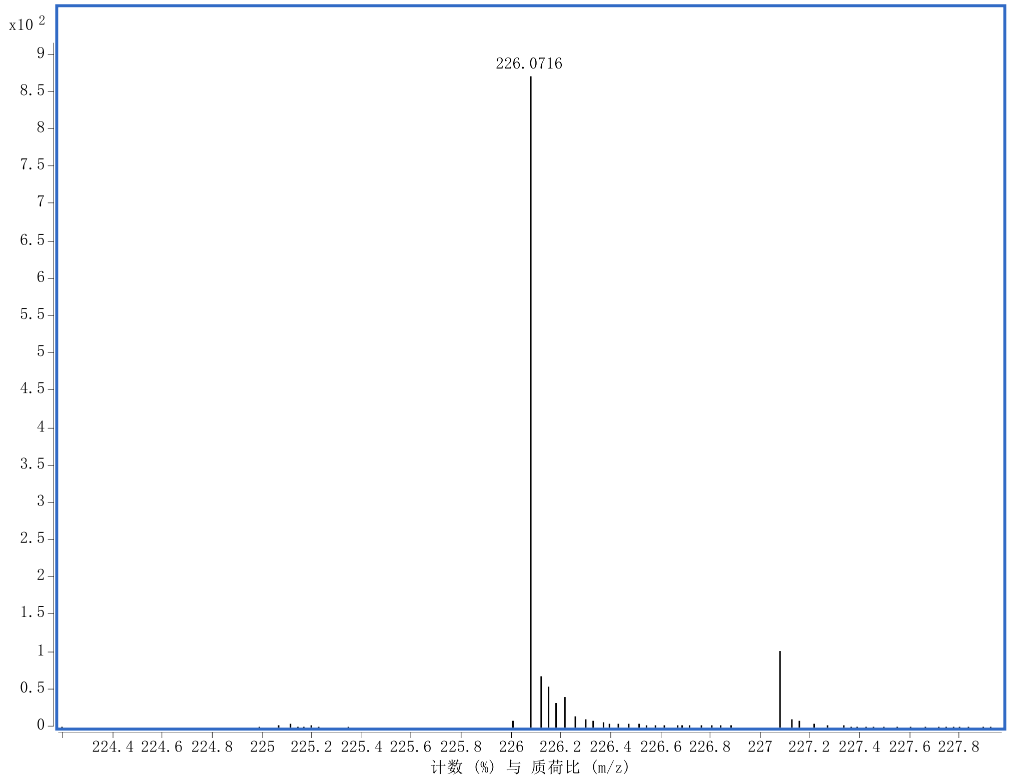

Figure S15. HRESIMS spectrum for **3**

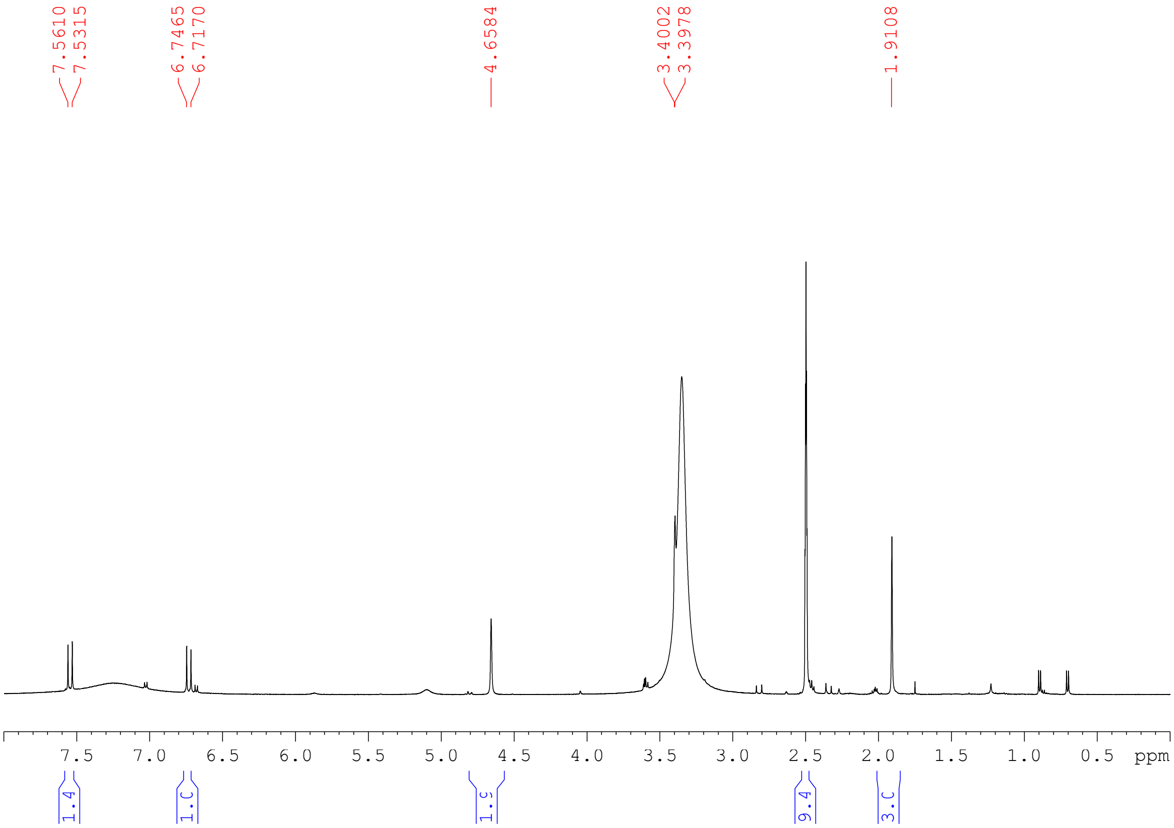

Figure S16. ^{1}H NMR spectrum (500 MHz, DMSO-d_6) of **3**

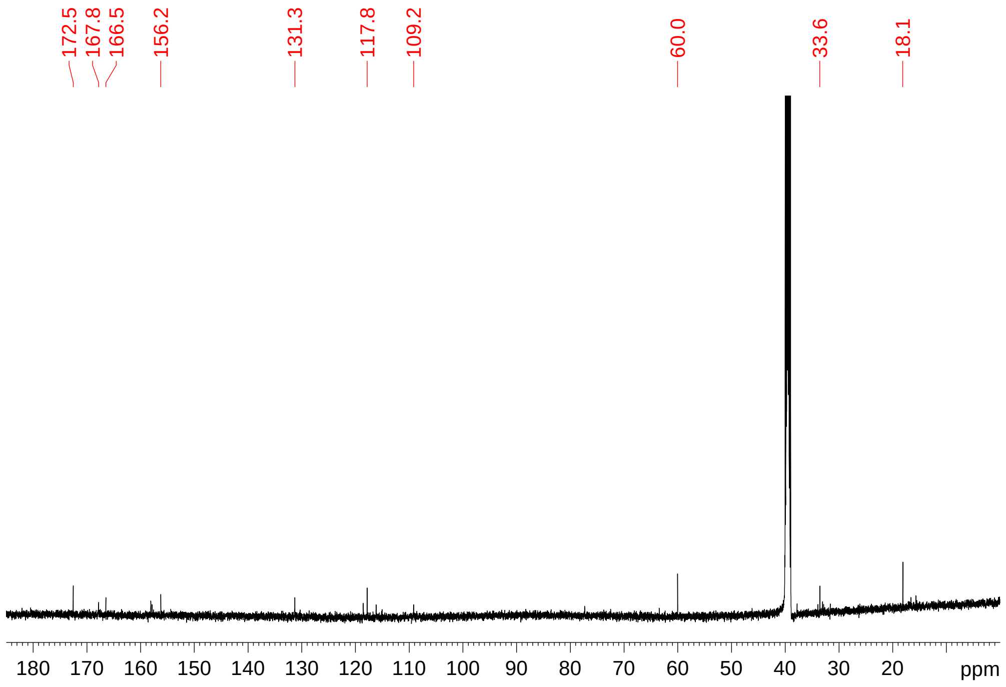

Figure S17. ^{13}C NMR spectrum (125 MHz, DMSO-d_6) of **3**

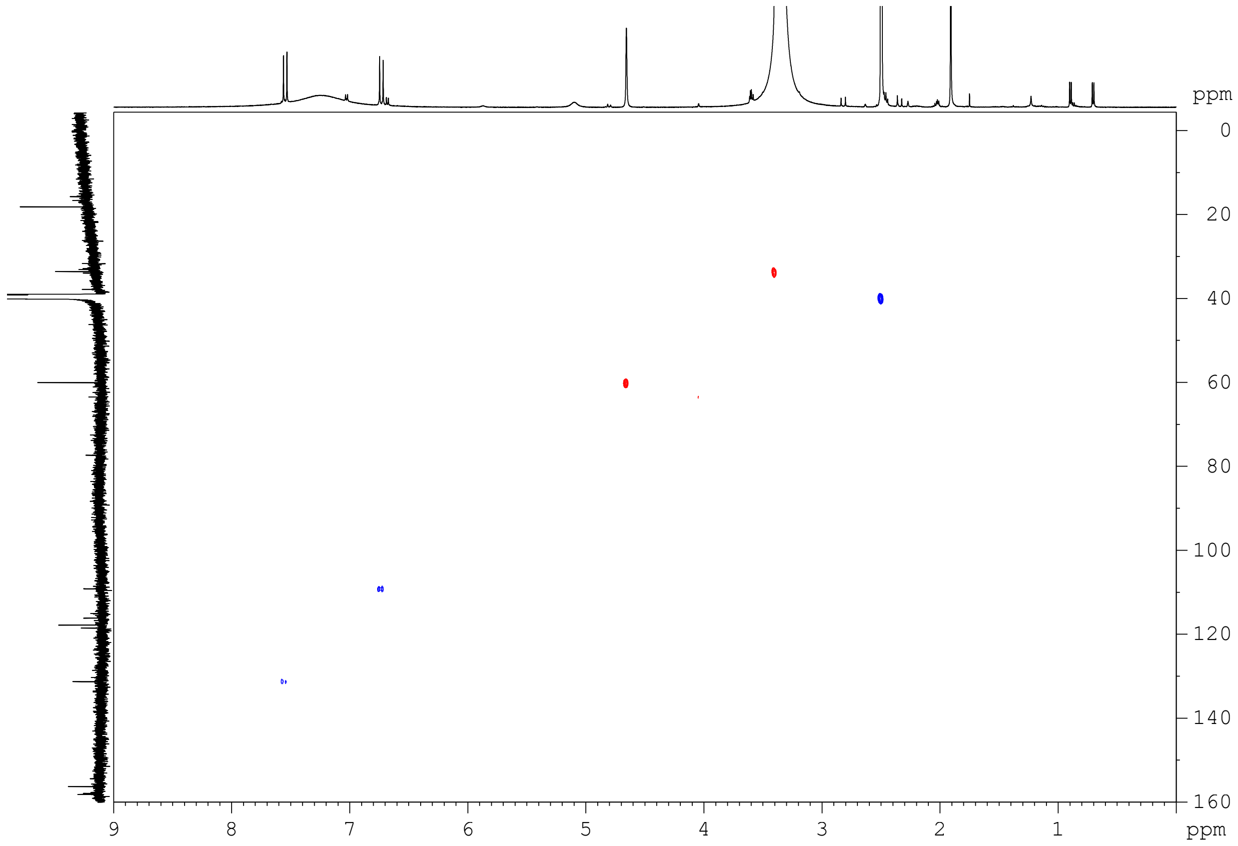

Figure S18. HSQC spectrum (500 MHz, DMSO-d_6) of **3**

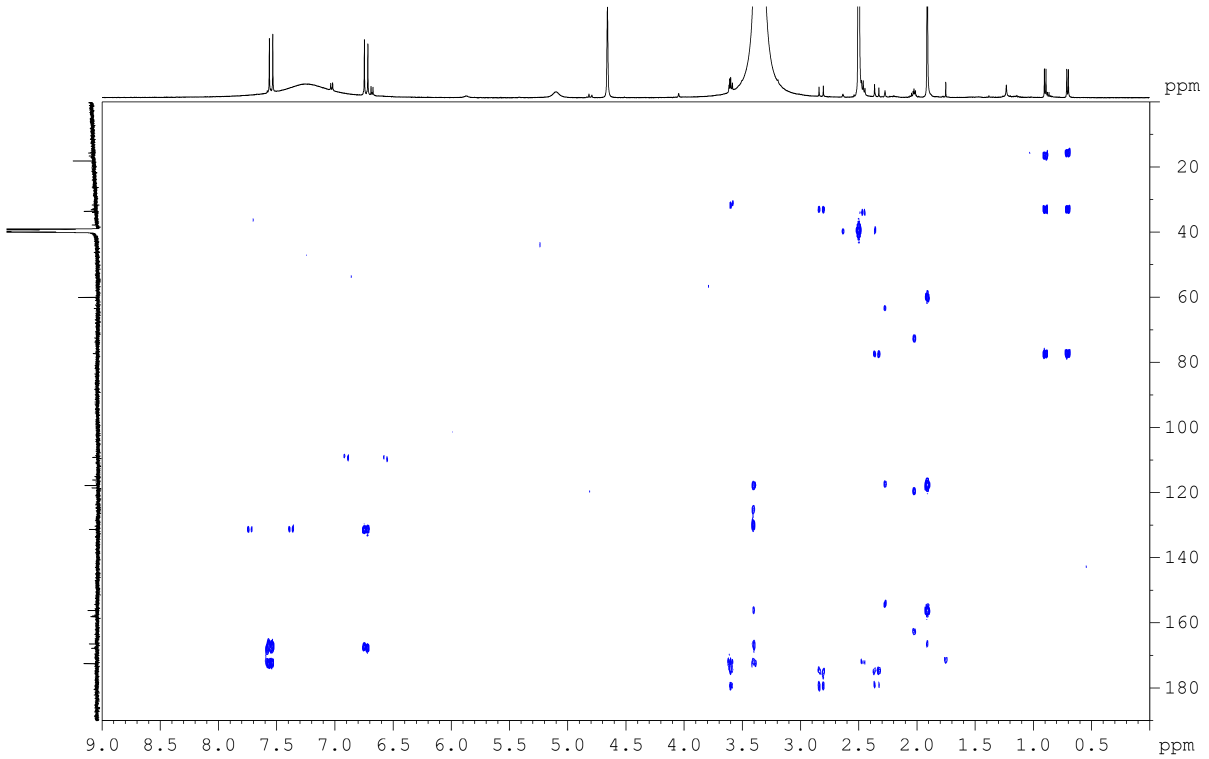

Figure S19. HMBC spectrum (500 MHz, DMSO-d_6) of **3**

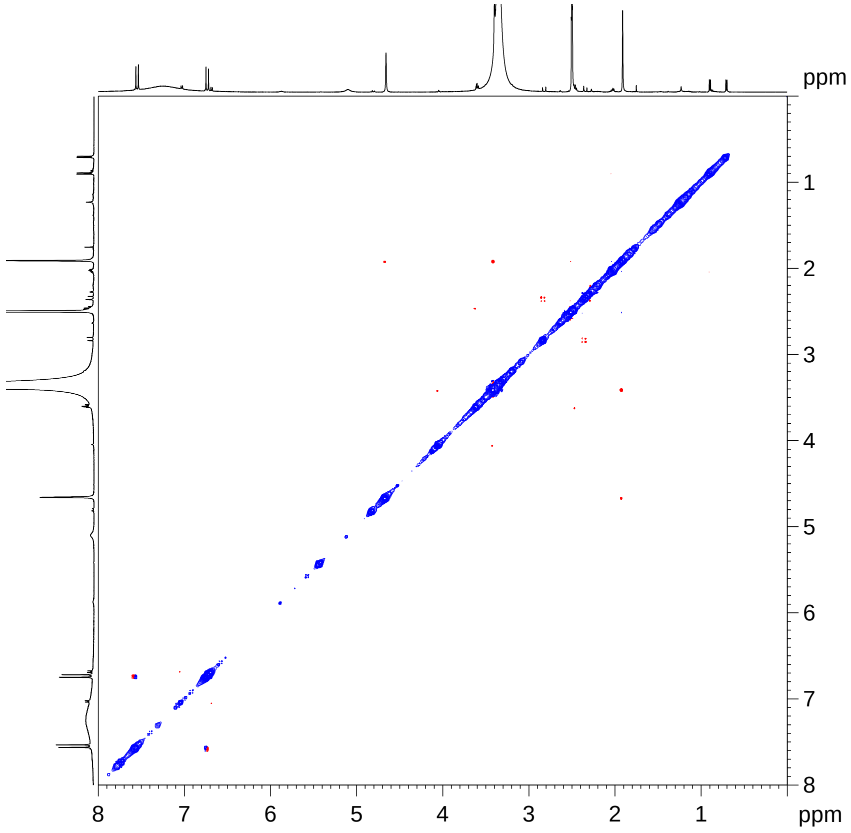

Figure S20. ROESY spectrum (500 MHz, DMSO-d_6) of **3**

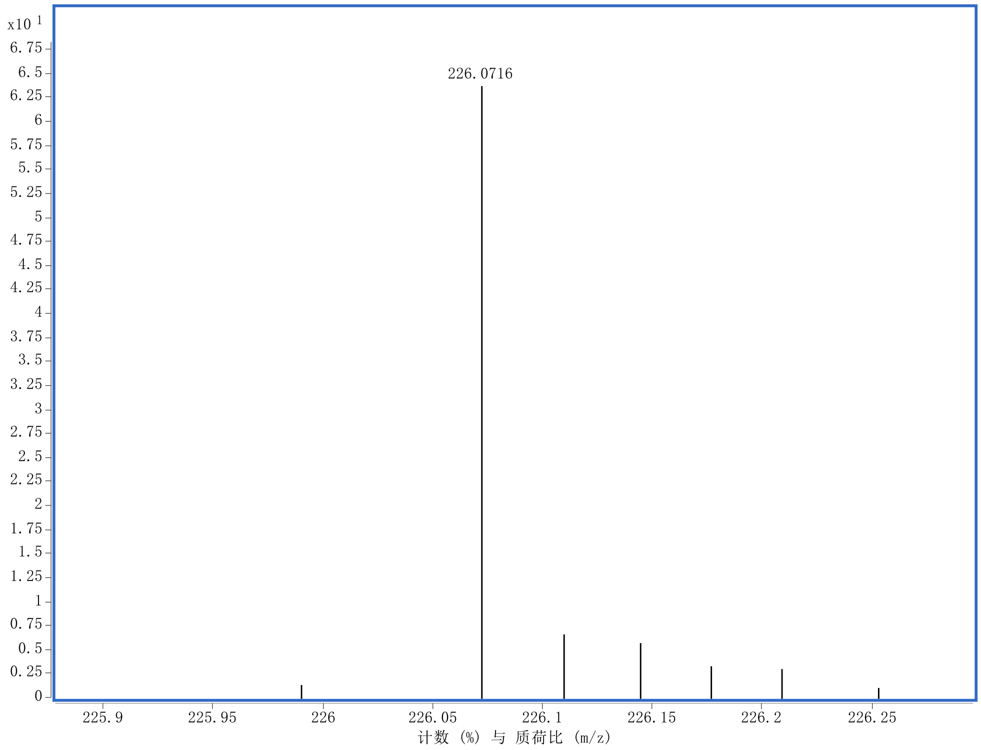

Figure S21. HRESIMS spectrum for **4**

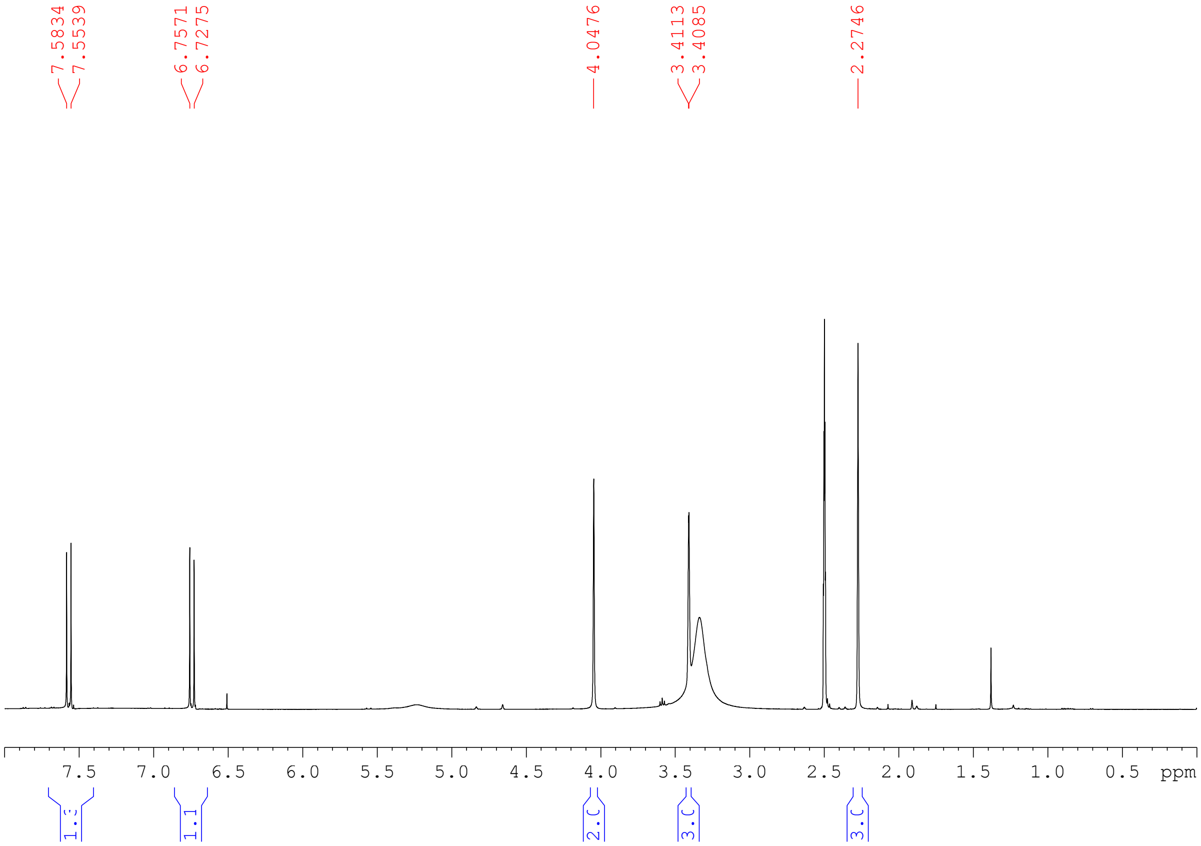

Figure S22. ^{1}H NMR spectrum (500 MHz, DMSO-d_6) of **4**

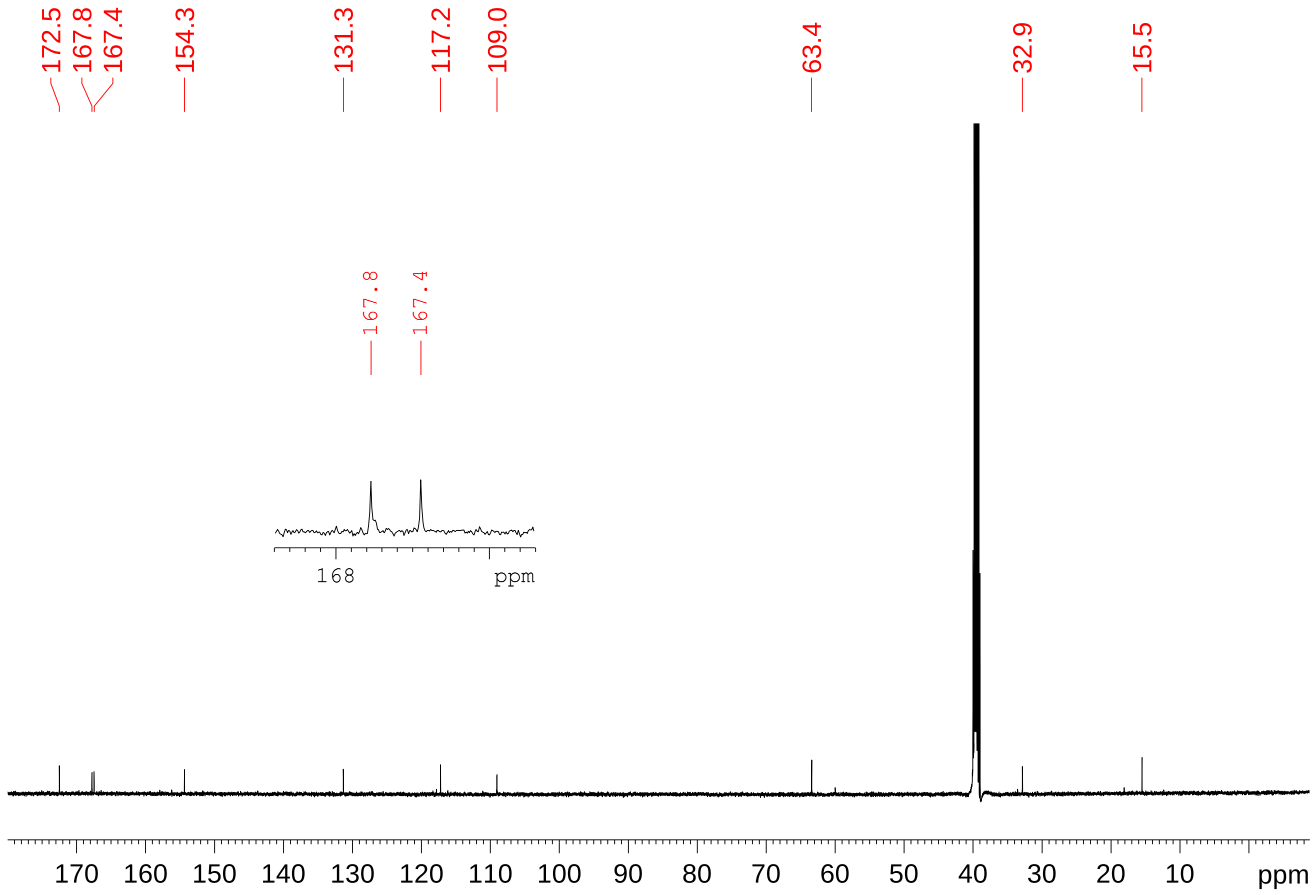

Figure S23. ^{13}C NMR spectrum (125 MHz, DMSO-d_6) of **4**

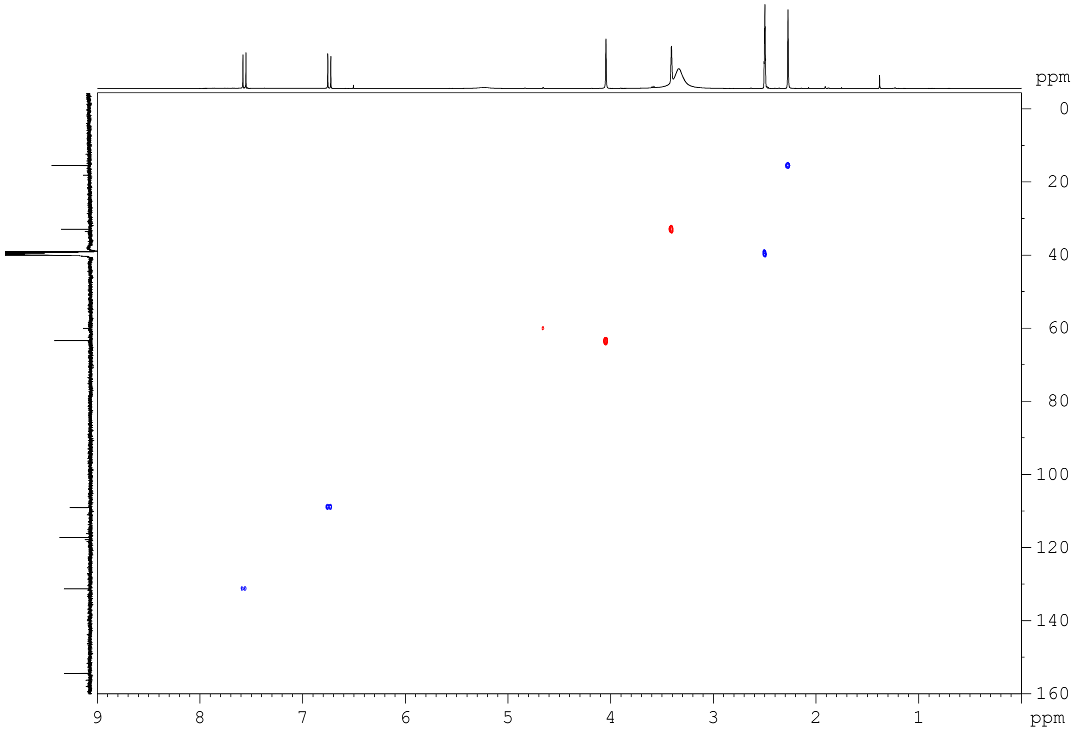

Figure S24. HSQC spectrum (500 MHz, DMSO-d_6) of **4**

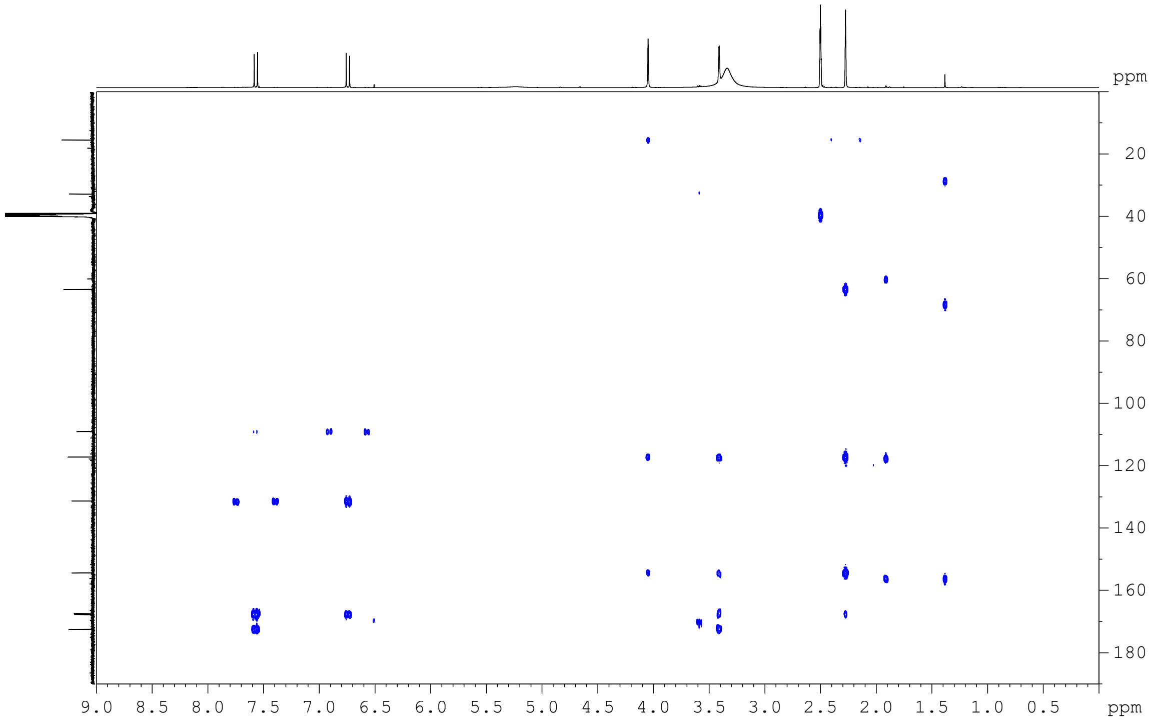

Figure S25. HMBC spectrum (500 MHz, DMSO-$_6$) of **4**

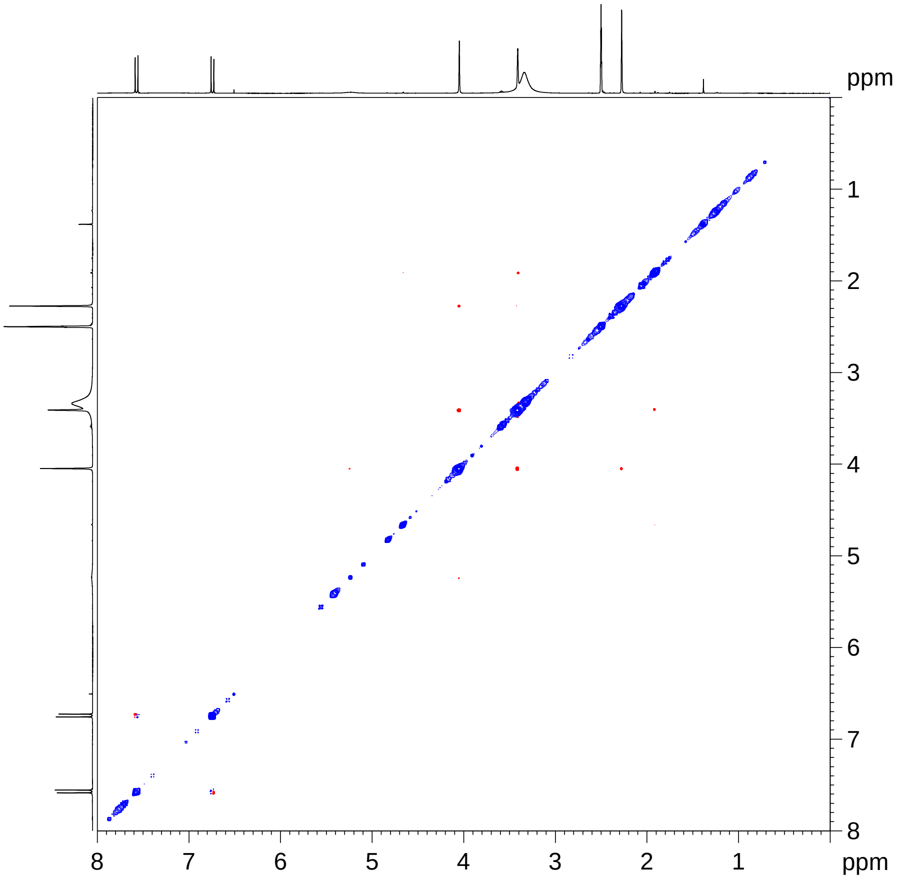

Figure S26. ROESY spectrum (500 MHz, DMSO-$_6$) of **4**

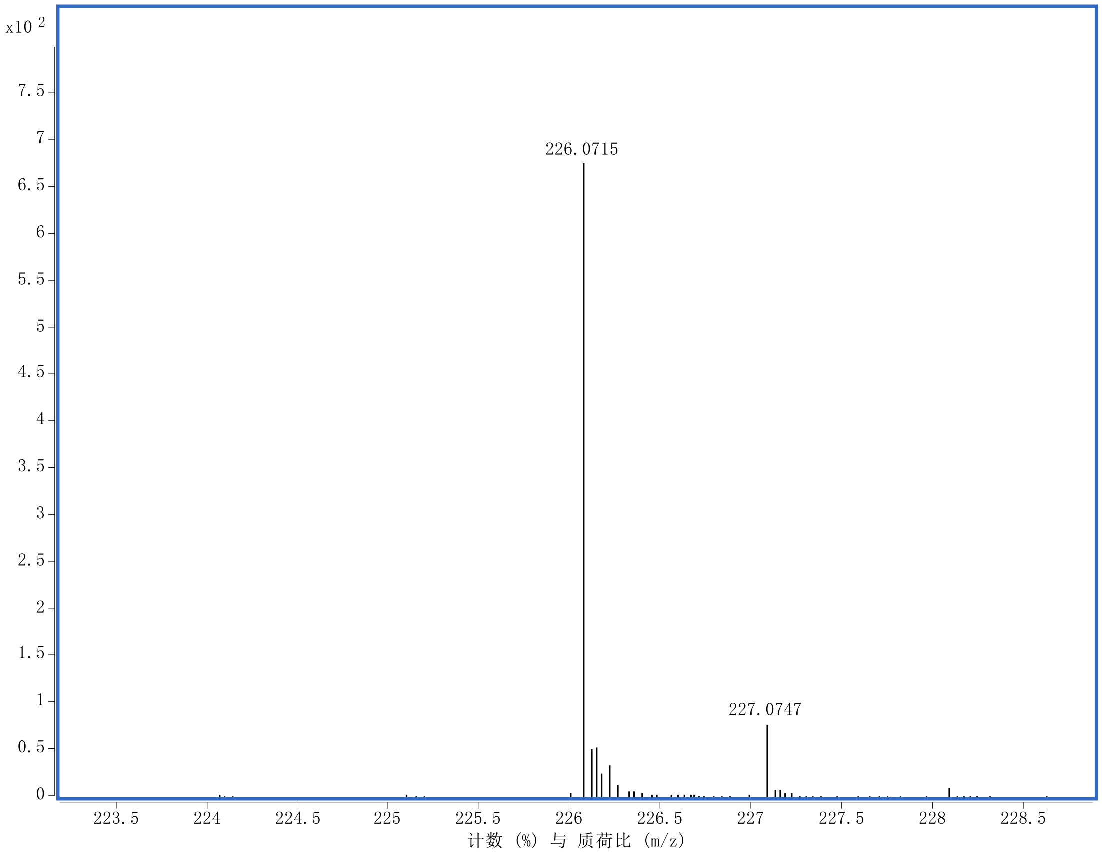

Figure S27. HRESIMS spectrum for **5**

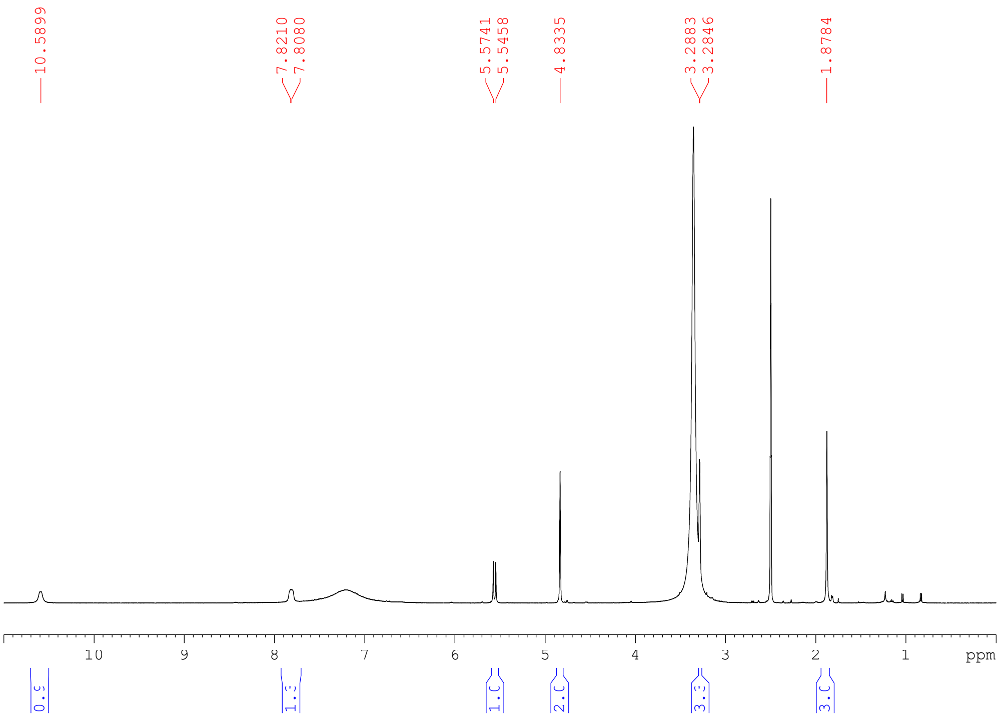

Figure S28. ^{1}H NMR spectrum (500 MHz, DMSO-d_6) of **5**

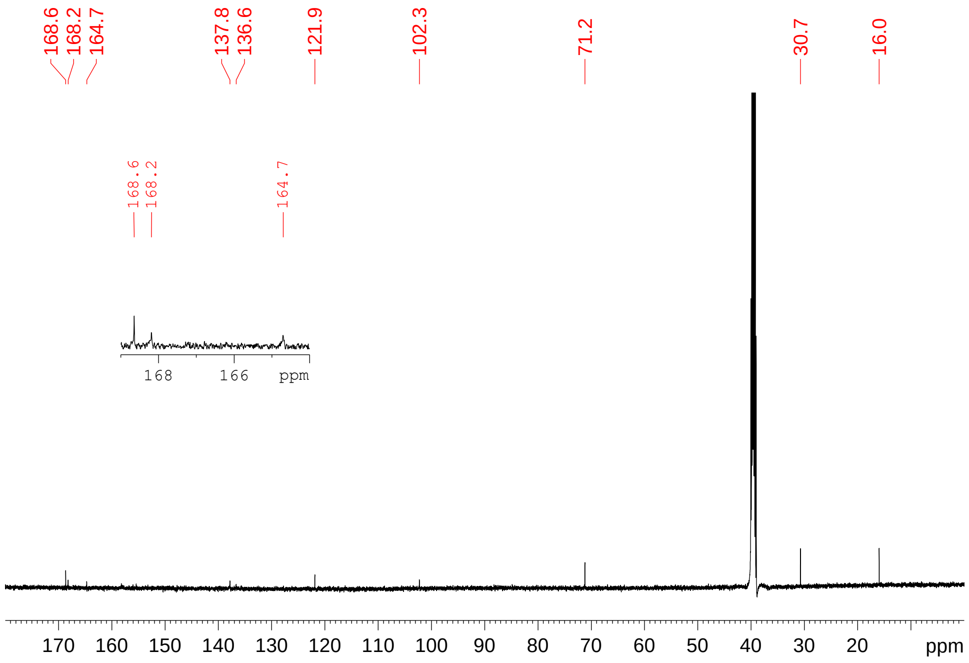

Figure S29. ^{13}C NMR spectrum (125 MHz, DMSO-d_6) of **5**

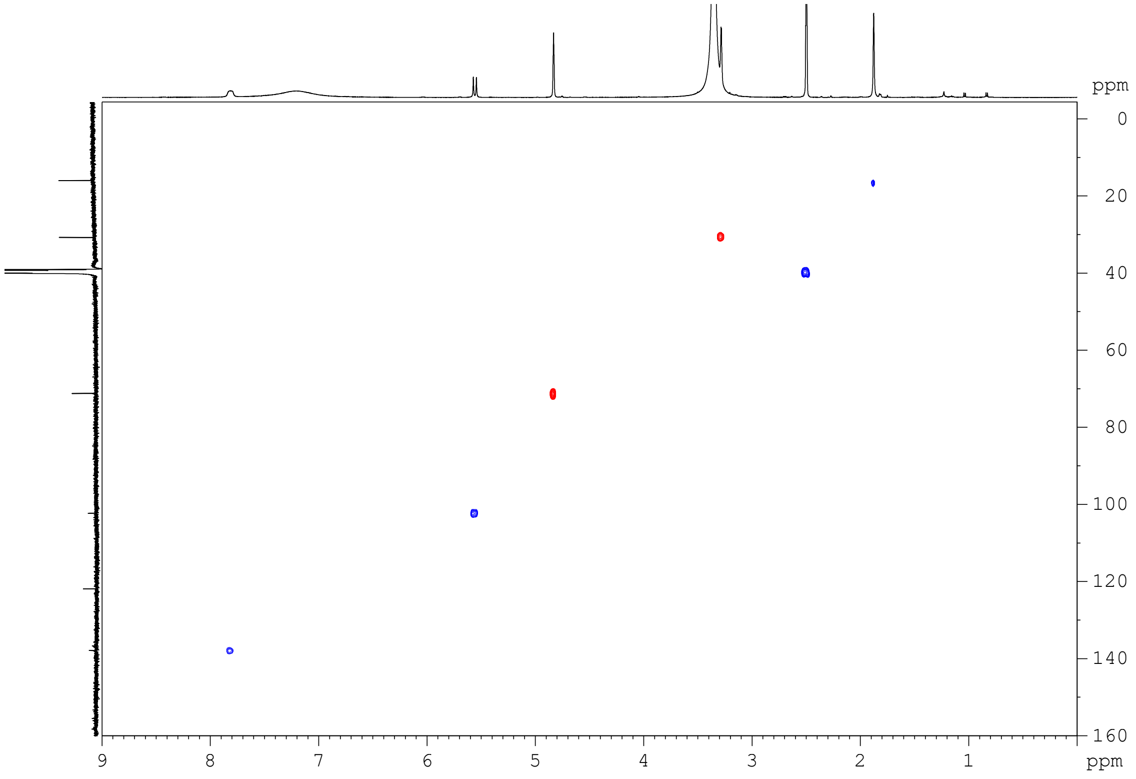

Figure S30. HSQC spectrum (500 MHz, DMSO-d_6) of **5**

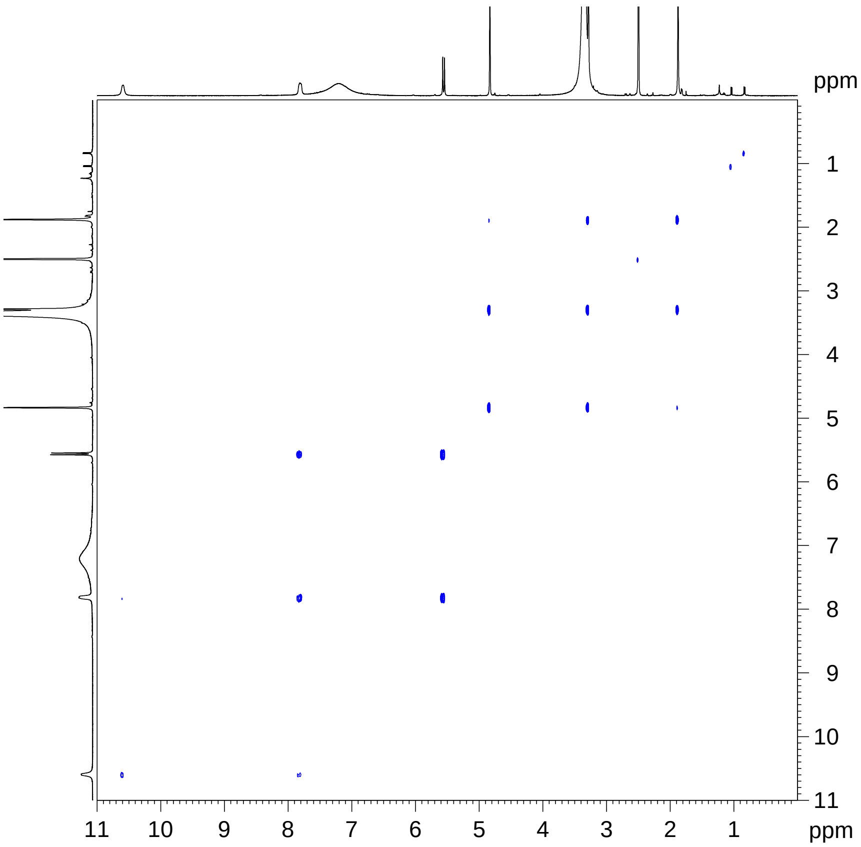

Figure S31. ^{1}H -^{1}H COSY spectrum (500 MHz, DMSO-d_6) of **5**

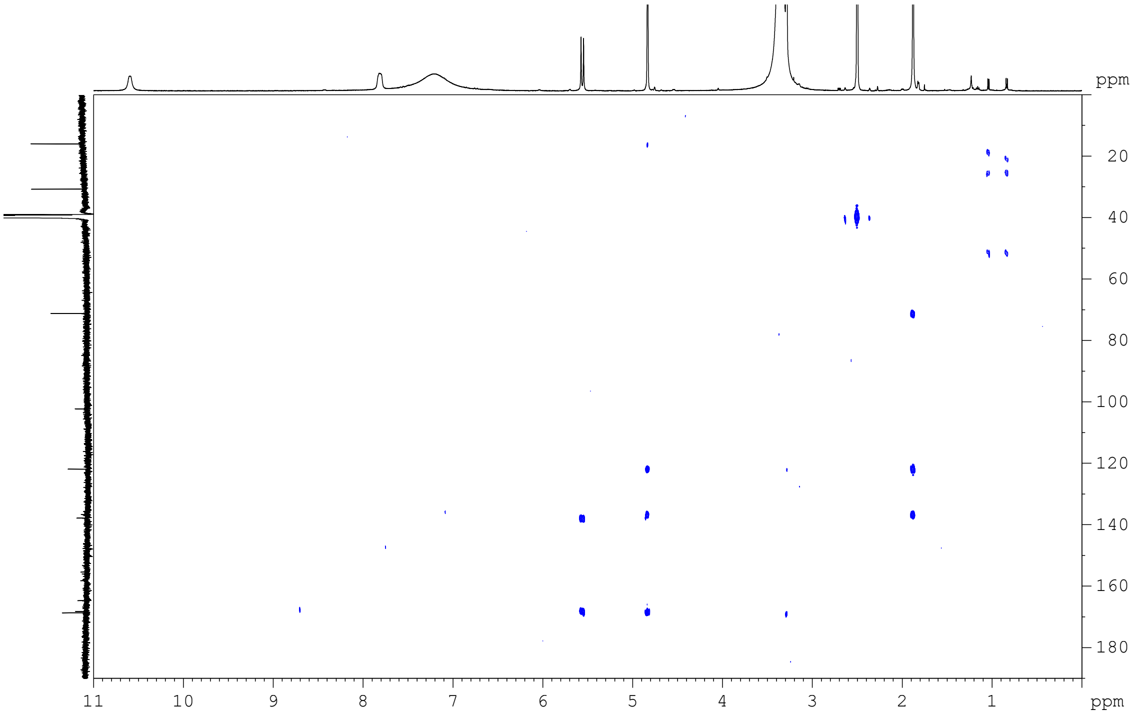

Figure S32. HMBC spectrum (500 MHz, DMSO-d_6) of **5**

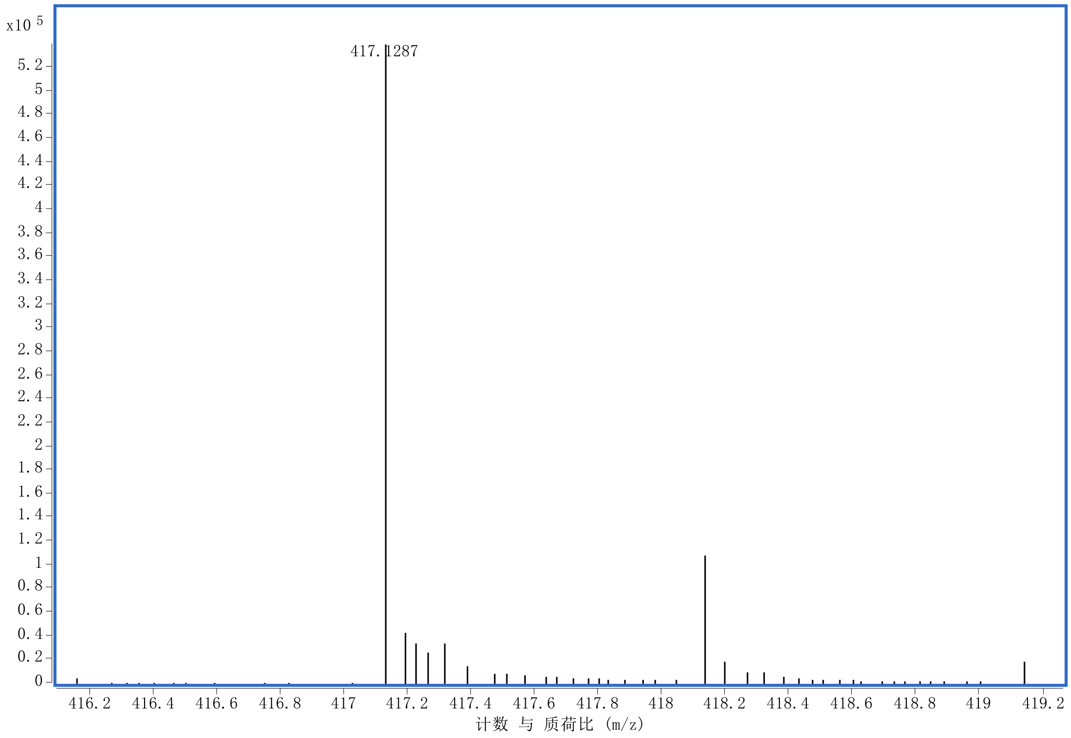

Figure S33. HRESIMS spectrum for **6**

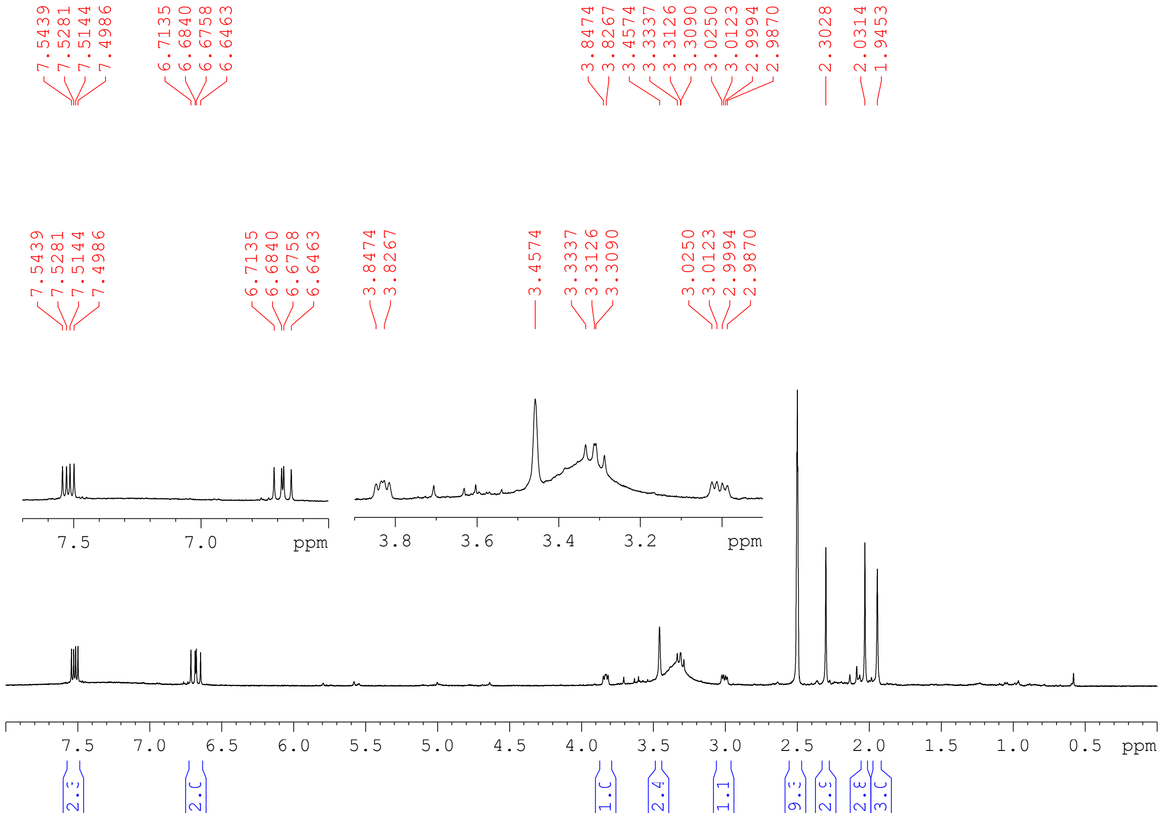

Figure S34. ^{1}H NMR spectrum (500 MHz, DMSO-d_6) of **6**

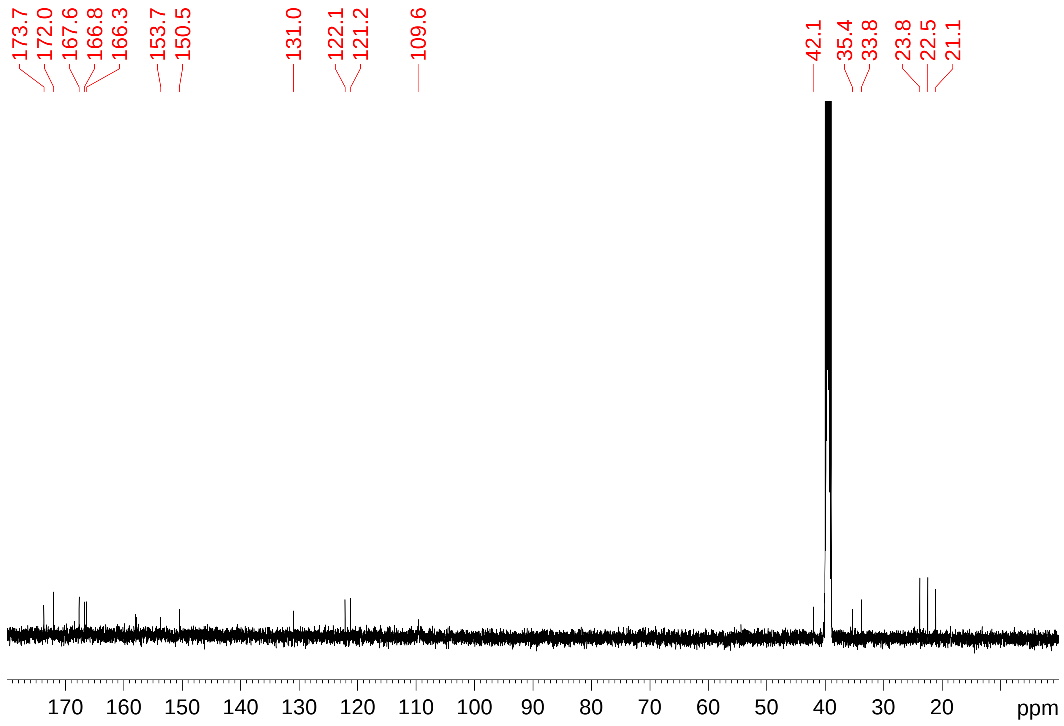

Figure S35. ^{13}C NMR spectrum (125 MHz, DMSO-d_6) of **6**

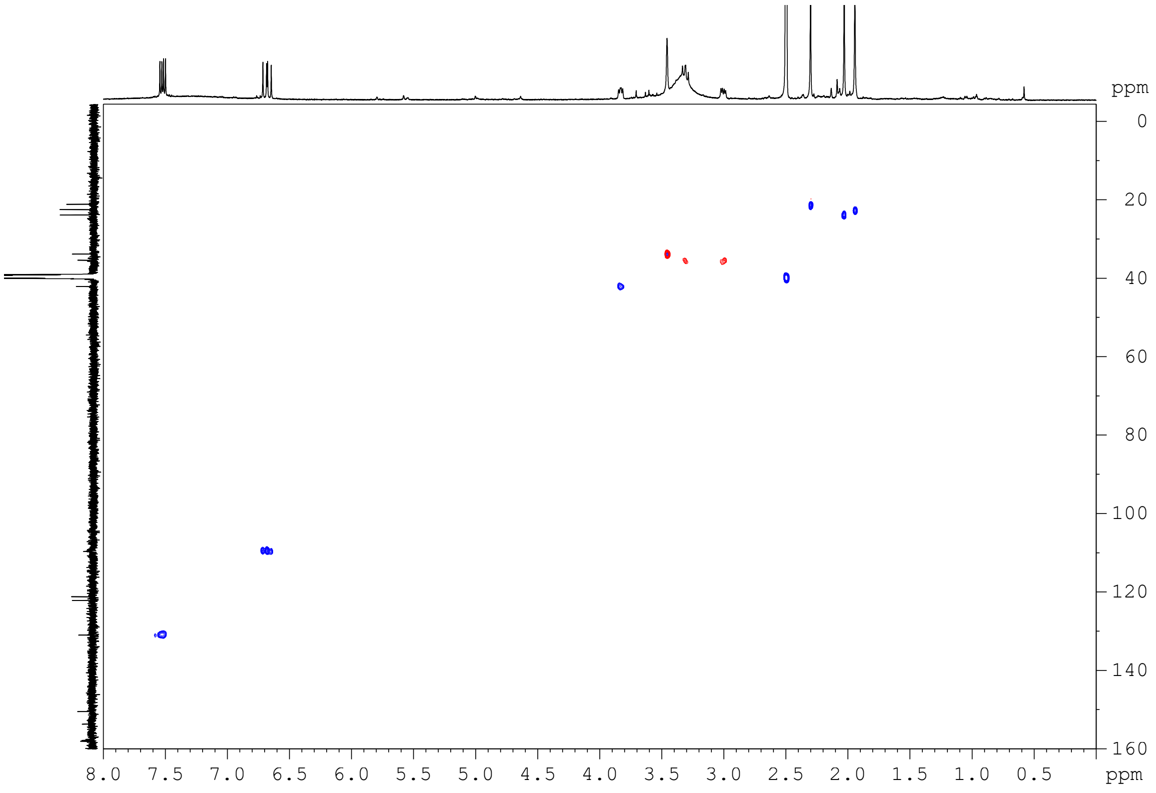

Figure S36. HSQC spectrum (500 MHz, DMSO-d_6) of **6**

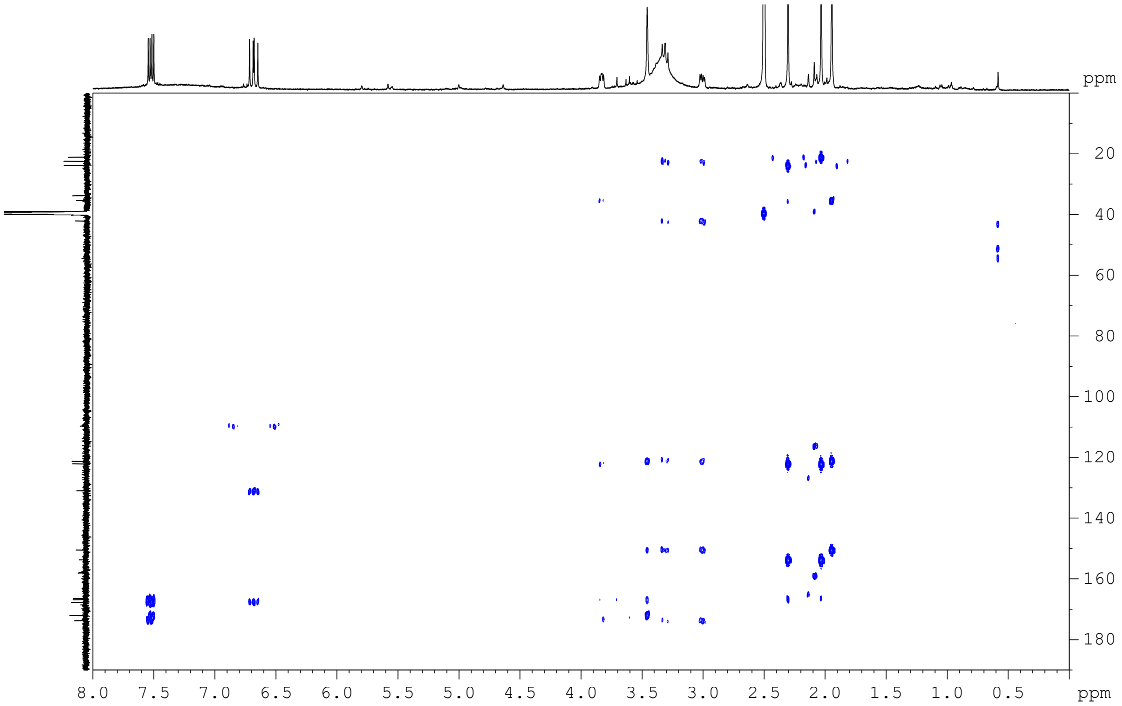

Figure S37. HMBC spectrum (500 MHz, DMSO-d_6) of **6**

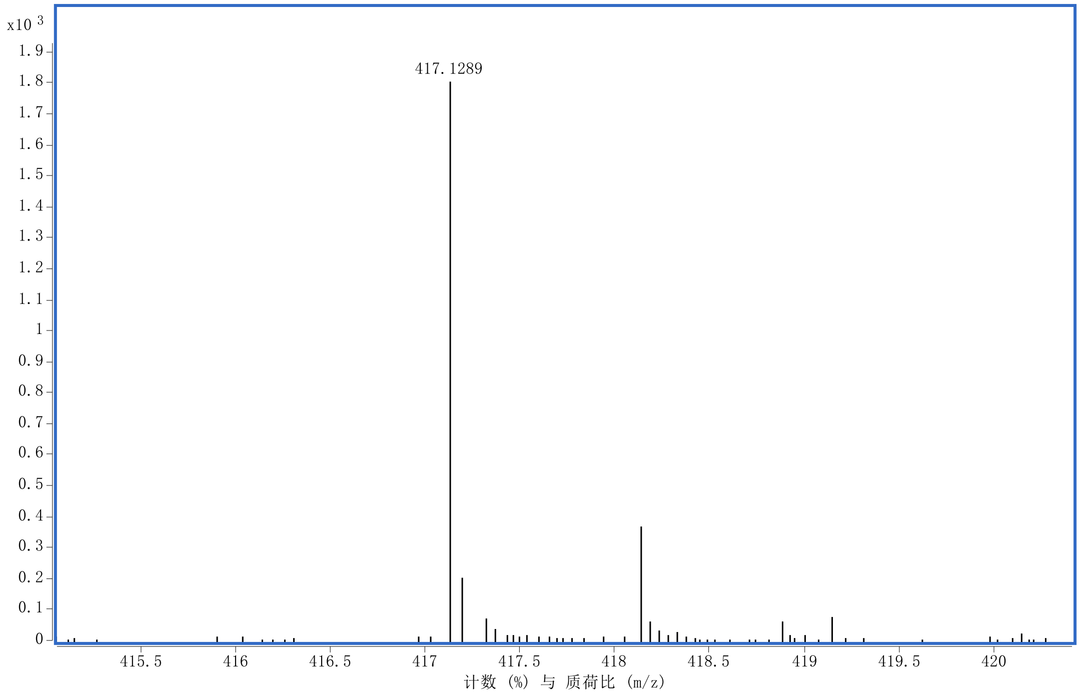

Figure S38. HRESIMS spectrum for **7**

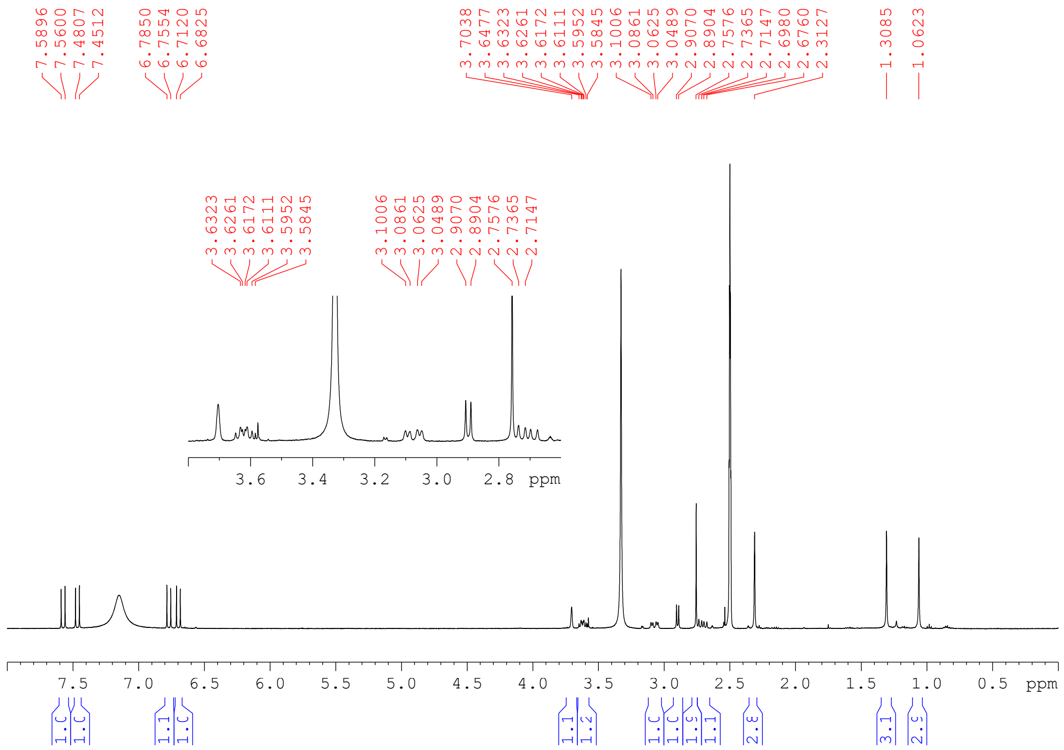

Figure S39. ^{1}H NMR spectrum (500 MHz, DMSO-d_6) of **7**

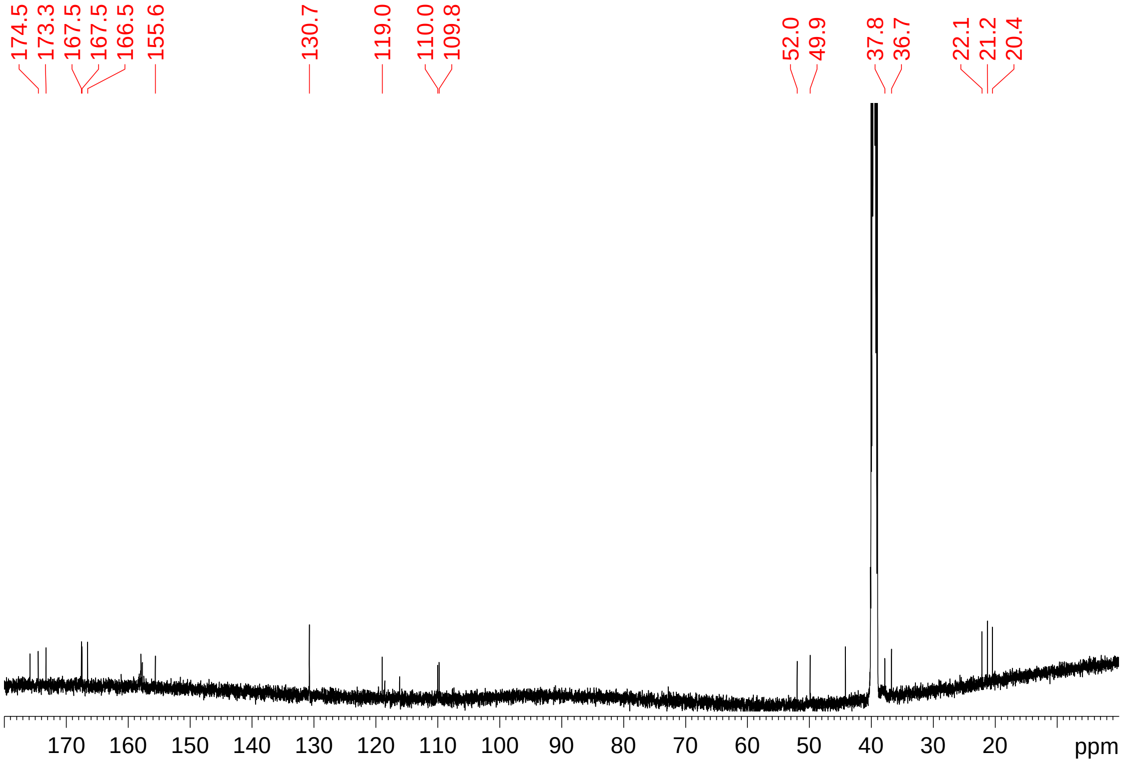

Figure S40. ^{13}C NMR spectrum (125 MHz, DMSO-d_6) of **7**

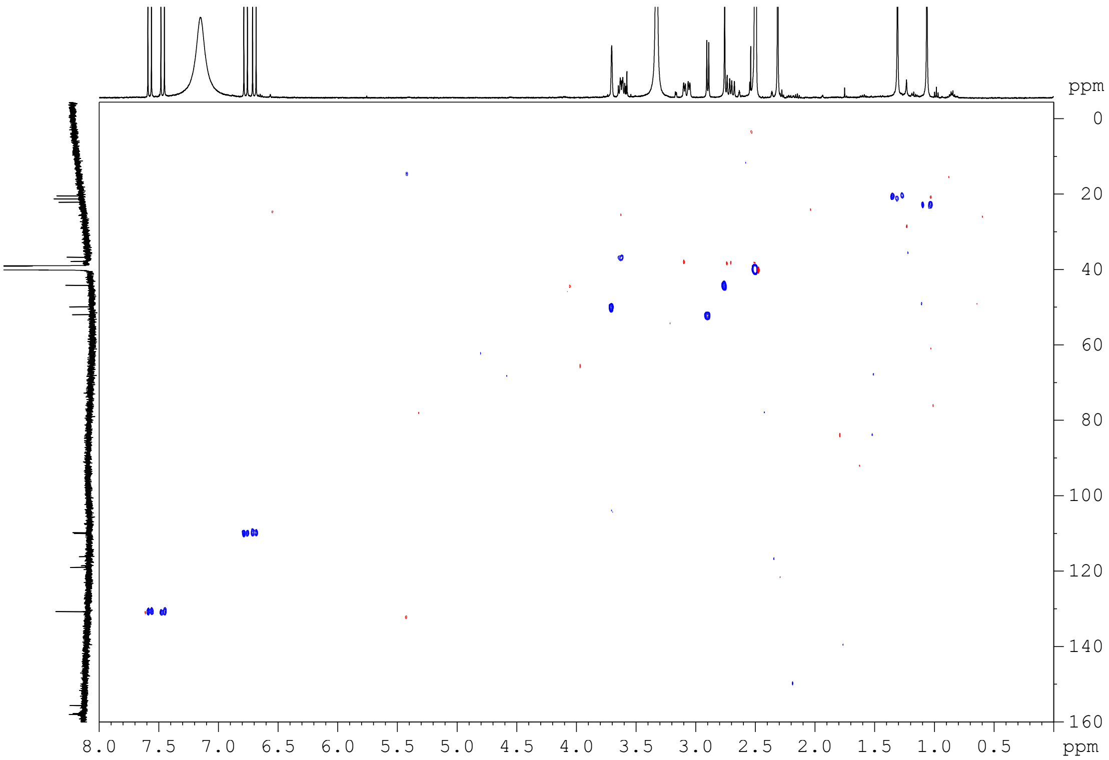

Figure S41. HSQC spectrum (500 MHz, DMSO-d_6) of **7**

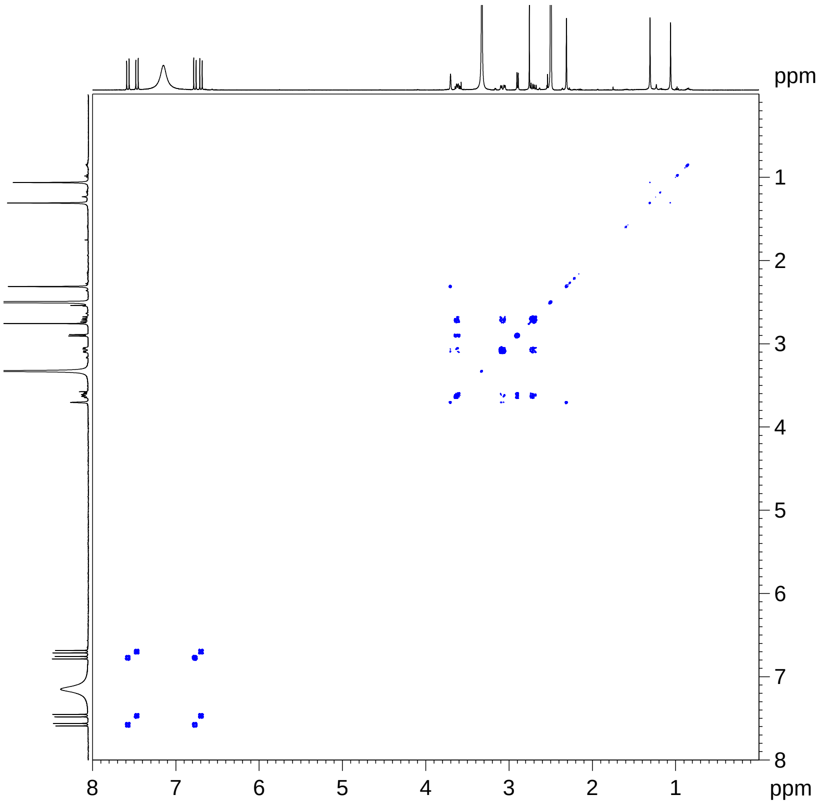

Figure S42. ^{1}H -^{1}H COSY spectrum (500 MHz, DMSO-d_6) of **12**

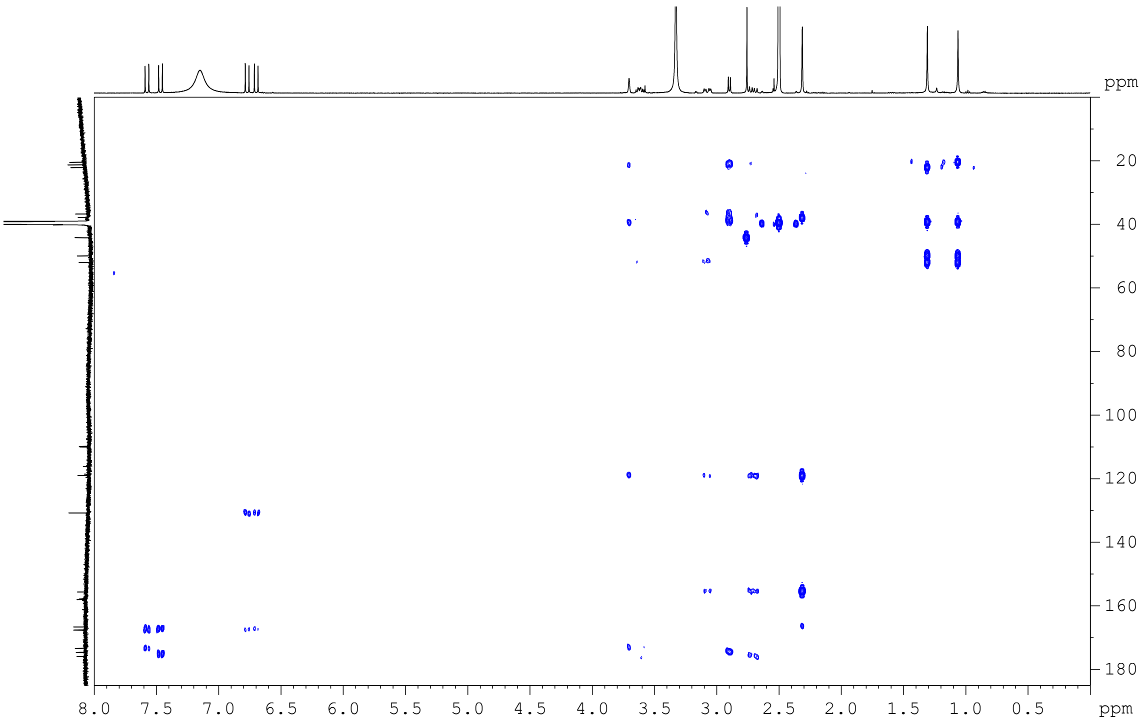

Figure S43. HMBC spectrum (500 MHz, DMSO-d_6) of **7**

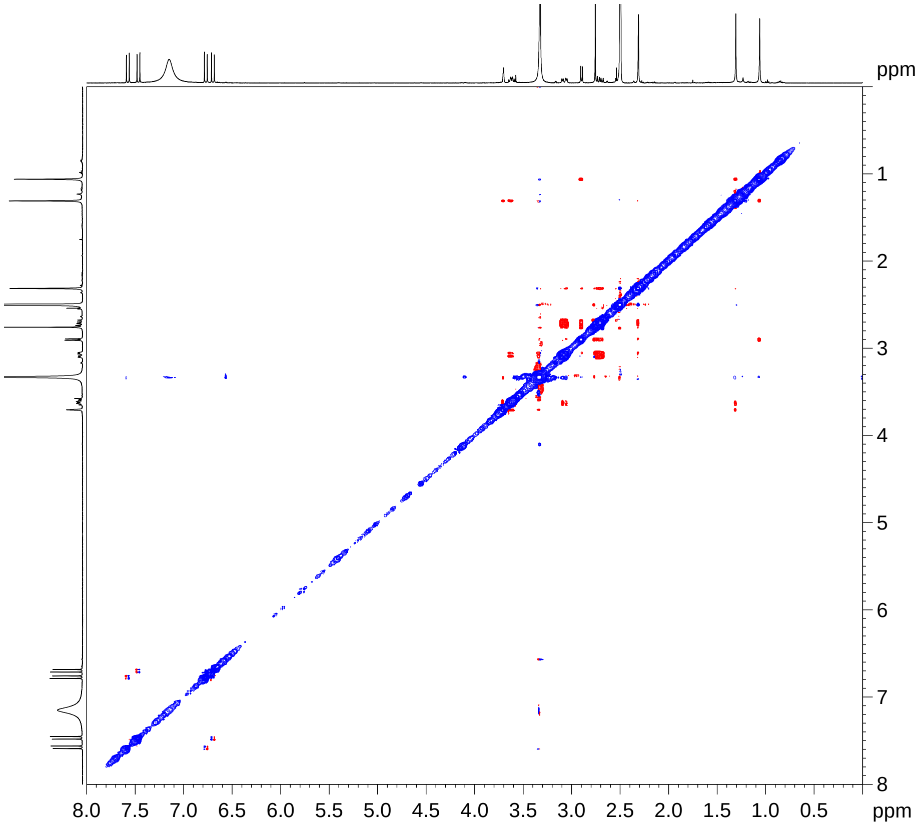

Figure S44. ROESY spectrum (500 MHz, DMSO-d_6) of **7**

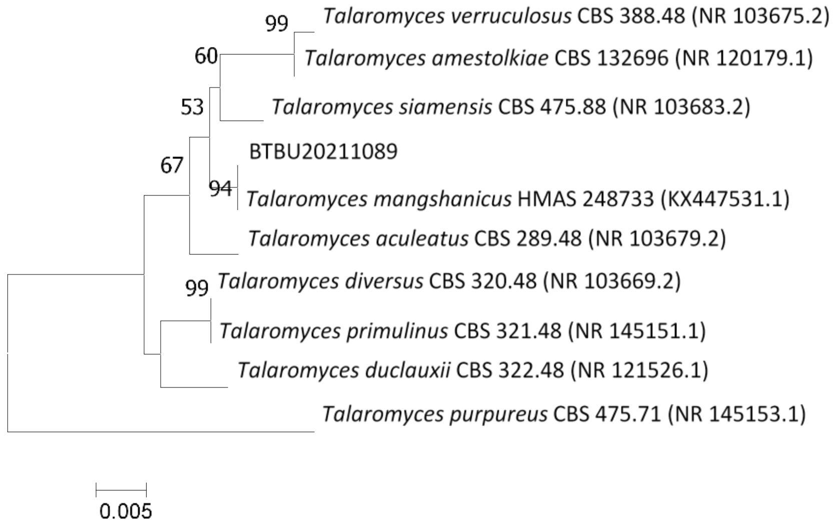

Figure S45. Neighbor-joining phylogenetic tree of BTBU20211089 and its most related type strains based on internal transcribed spacer region (ITS) from NCBI ITS database. Numbers at nodes indicate levels of bootstrap support (%) based on a neighbor joining analysis of 1,000 resampled datasets; only values >50 % are given. NCBI accession numbers are given in parentheses. Bar 0.005 nucleotide substitutions per site.
